# Investigation of Genes Encoding Calcineurin B-Like Protein Family in Legumes and Their Expression Analyses in Chickpea (*Cicer arietinum* L.)

**DOI:** 10.1371/journal.pone.0123640

**Published:** 2015-04-08

**Authors:** Mukesh Kumar Meena, Sanjay Ghawana, Atish Sardar, Vikas Dwivedi, Hitaishi Khandal, Riti Roy, Debasis Chattopadhyay

**Affiliations:** National Institute of Plant Genome Research, Aruna Asaf Ali Marg, New Delhi, 110067, India; National Institute of Plant Genome Research, INDIA

## Abstract

Calcium ion (Ca^2+^) is a ubiquitous second messenger that transmits various internal and external signals including stresses and, therefore, is important for plants’ response process. Calcineurin B-like proteins (CBLs) are one of the plant calcium sensors, which sense and convey the changes in cytosolic Ca^2+^-concentration for response process. A search in four leguminous plant (soybean, *Medicago truncatula*, common bean and chickpea) genomes identified 9 to 15 genes in each species that encode CBL proteins. Sequence analyses of CBL peptides and coding sequences (CDS) suggested that there are nine original CBL genes in these legumes and some of them were multiplied during whole genome or local gene duplication. Coding sequences of chickpea CBL genes (*CaCBL*) were cloned from their cDNAs and sequenced, and their annotations in the genome assemblies were corrected accordingly. Analyses of protein sequences and gene structures of CBL family in plant kingdom indicated its diverse origin but showed a remarkable conservation in overall protein structure with appearance of complex gene structure in the course of evolution. Expression of *CaCBL* genes in different tissues and in response to different stress and hormone treatment were studied. Most of the *CaCBL* genes exhibited high expression in flowers. Expression profile of *CaCBL* genes in response to different abiotic stresses and hormones related to development and stresses (ABA, auxin, cytokinin, SA and JA) at different time intervals suggests their diverse roles in development and plant defence in addition to abiotic stress tolerance. These data not only contribute to a better understanding of the complex regulation of chickpea CBL gene family, but also provide valuable information for further research in chickpea functional genomics.

## Introduction

External and internal stimuli cause change in cytosolic calcium ion (Ca^2+^) concentration. Kinetics and magnitude of calcium ion concentration, i.e. ‘calcium signature’ varies with signals and possibly contributes to the specificity of response [1]. Various calcium sensor proteins in plants including recently identified calcineurin B-like proteins (CBLs) recognise and transmit these specific signals. CBLs exhibit significant similarity to regulatory subunit B of calcineurin (CNB) of *Saccharomyces cerevisiae* and animal neuronal calcium sensor (NCS) protein. CBL proteins possess four EF-hand domains for binding a maximum of four Ca^2+^ ions at a time. They mediate calcium signaling by binding to and activating protein kinases named CBL interacting protein kinases (CIPKs), which are similar to sucrose nonfermenting (SNF) protein kinases in yeast and AMP dependent kinases (AMPKs) in animals in their kinase domains [2].

So far, 10 CBLs and 26 CIPKs in *Arabidopsis* (*Arabidopsis thaliana*) and 10 CBLs and 31 CIPKs in rice were identified with the help of genetic and bioinformatic analyses [3–5]. CBLs were first reported in model plant *Arabidopsis* in a genetic screening of *salt overly sensitive (sos)* mutants. *SOS3* gene was identified as a member of CBL family and referred to as *CBL4* [6]. AtCBL4/SOS3 plays an important role in SOS pathway by interacting with SOS2 or AtCIPK24. AtCBL4-AtCIPK6 complex modulates activity of potassium (K^+^) channel AKT2 by mediating its translocation from endoplasmic reticulum membrane to plasma membrane [7]. AtCBL10 and AtCIPK24 together were reported to regulate Na^+^-homeostasis in vacuolar membrane indirectly by regulating unknown targets in shoot and leaves [8]. AtCBL10 directly interacts with AKT1 to regulate K^+^ homeostasis without binding to any CIPKs [9]. AtCBL1 expression was shown to be induced by drought, cold and wounding [10], but not by abscisic acid (ABA) [11]. Studies with loss-of-function mutant of AtCBL1 supported its role in several abiotic stress responses in an ABA independent manner [10, 11]. Loss of function mutant of AtCBL9 (closely related to AtCBL1) was found to be hypersensitive to ABA [12]. AtCBL1-AtCIPK1 complex is involved in ABA-dependent stress response whereas the AtCBL9-AtCIPK1 complex plays role in ABA-independent stress response [13]. Recent studies report that AtCBL1 responds to glucose and gibberellin (GA) signals during germination and seedling development [14]. Another study showed that AtCBL1/9 forms complex with AtCIPK23 and regulates K^+^ and nitrate (NO_3_^-^) uptake in plants [15–17]. The vacuolar membrane localized proteins AtCBL2 and AtCBL3 regulate V-ATPase activity, thus regulating intracellular ion homeostasis [18].

*Arabidopsis* CBL proteins were structurally divided into two groups according to length of their N-terminal domains; CBL proteins with a short N-terminal domain of 27–32 amino acids and CBL proteins with an extended N-terminus of 41–43 amino acids. The short N-terminus-containing group comprises of CBL1, CBL4, CBL5, CBL8 and CBL9. Except CBL8, all harbor conserved motifs for lipid modification in the N-terminus [19]. CBL1, CBL4, CBL5 and CBL9 were shown to be myristoylated at their N-terminus [20]. CBL2, CBL3, CBL6, CBL7 and CBL10 constitute the second group. These proteins harbor an extended N-terminal domain that is similar to the K^+^-channel interacting proteins (KChIP) from NCS group and does not contain discernible lipid modification motifs. AtCBL10 is special in this group in that it harbors a very long N-terminal extension forming a potential single transmembrane domain important for its localization [8, 21].

Like other gene families, CBL genes have mostly been studied in *Arabidopsis* and information on CBL genes from other plants is limited. Overexpression of soybean (*Glycine max*) CBL1 (GmCBL1), an ortholog of AtCBL1, enhances tolerance to salinity and drought stress in *Arabidopsis* [22]. CBL3 from chickpea (*Cicer arietinum*) was shown to interact with CIPK6 and mediate response to dehydration and salinity stresses [23]. PeCBL1 interacts with CIPK24, CIPK25 and CIPK26 to regulate Na^+^/K^+^ homeostasis in *Populus euphratica* [24]. In *Brassica napus*, BnaCBL1-CIPK6 complex mediates plant response to high salinity, phosphorous deficiency and ABA signaling [25]. Grapevine (*Vitis vinifera*) VvCBL1-VvCIPK4 and VvCBL2-VvCIPK3 complexes control activity of K^+^ channel [26]. CBL proteins also play role in plant immunity. Tomato CBL10 and CIPK6 interact with respiratory burst homolog B protein (RbohB) at the plasma membrane and contribute to ROS generation during effector-triggered immunity [27]. From all the studies mentioned above and from the numbers of CBLs and CIPKs in a species, it is evident that a particular CBL can interact with and activate multiple CIPKs depending upon circumstances and CBL-CIPK pathways are essential for important cellular processes mostly for stress signaling.

Legumes are important in plant kingdom because of their uniqueness in fixing atmospheric nitrogen with help of nitrogen-fixing bacteria. In this report, we identified CBL-encoding genes in the genome-assemblies of four legumes namely, soybean, *Medicago truncatula*, common bean (*Phaseolus vulgaris*) and chickpea. We have analysed peptide sequences and gene structures of CBL proteins from these leguminous plants with reference to those from plants with evolutionary significance. Subsequently, we performed expression profiling of chickpea CBL genes in different tissues and in response to different stresses and hormone treatments at different time points, and discussed their potential roles in different cellular processes.

## Materials and Methods

### Identification of CBL gene family members

Chickpea *CBL* genes were identified using Chickpea Transcriptome Database, [28] and genome annotation [29, 30]. *Arabidopsis* and rice CBL peptide sequences were used as queries to find out orthologous CBL genes by BLASTP. The peptide models were designated as *CaCBL*s (*C*. *arietinum* CBL) and named according to their sequence similarity with the corresponding *Arabidopsis* CBLs. Further, *Arabidopsis* CBL gene sequences were used to search both the chickpea genome sequences to find out similar gene sequences. *CaCBL* gene sequences identified in one chickpea variety were used to search similar sequences in the genome of the other chickpea variety. *CBL* genes in three legumes, *Medicago*, soybean and common bean and the moss *Physcomitrella patens* were identified by searching annotated gene models in Phytozome (http://www.phytozome.net/) and EST sequences in NCBI (http://www.ncbi.nlm.nih.gov/) databases; and for *Ostreococcus tauri* and *Chlorella variabilis* in NCBI database only using keyword ‘calcineurin B’ and BLASTP searches with *Arabidopsis* and rice CBL peptide sequences as queries. Identifiers of these genes were mentioned as per their Phytozome annotation. Identifier of CBL from *Ostreococcus* was mentioned according to its GenBank accession number as this was not available in Phytozome. Coding sequences (CDS) of all 9 predicted *CaCBL* genes were amplified from cDNA template, cloned and sequenced from both the ends using ABI3730XL sequencer (Applied Biosystems, Foster City, CA, USA). The primers used for cloning were designed using 5’- and 3’-sequences of the predicted translation start and stop codons, respectively (S1 Table).

### Mapping of *CaCBL* genes on chromosomes and gene structure analysis

To determine the location of *CaCBL* genes on chromosome, the genomic sequences of *CaCBL* were used as query sequences for the BLASTN search against sequenced chickpea genome. All 9 *CaCBL* genes were mapped on chromosomes according to BLASTN results. The exon-intron structures of *CaCBL* genes were determined by aligning their cDNA sequences to the corresponding genomic sequences using Gene Structure Display Server 2.0 program [31].

### Phylogenetic analysis of CBL proteins and protein structure modeling

Phylogenetic analysis of CBL peptide sequences was performed using MEGAv6 [32] by the neighbour-joining (NJ) method [33]. CBL protein sequences from different plant species *Arabidopsis*, chickpea, *Medicago*, soybean, common bean, *Ostreococcus*, *Chlorella* and *Physcomitrella* were retrieved from NCBI and Phytozome databases and aligned using ClustalX [34]. The reliability of phylogenetic tree was tested by bootstrap for 1000 replicates. Protein structure of CBL1 from *Arabidopsis*, chickpea, *Ostreococcus*, *Chlorella*, *Physcomitrella* and calcineurin B of yeast were generated using Phyre2 remote homology modeling server [35]. All five CBL structure from different plant species were analysed by pyMOL (PyMOL Molecular Graphics System, Version 1.1 Schrodinger, LLC). Rate of synonymous substitution (Ks) was calculated by PAL2NAL program [36] and period of divergence was calculated using the equation T = Ks/2r, where r was taken as 6.1X10^-9^ [37].

### Plant materials, growth condition and treatments

A desi-type drought tolerant [38] chickpea (*Cicer arietinum* L. cv. PUSABGD72) was used for gene expression study. Chickpea seedlings were grown in composite soil (3 part agropeat + 1 part vermiculite) under suitable greenhouse condition (temperature 23–25°C and photo period 14hrs light/10hrs dark). 6 day-old seedlings were exposed to 250mM NaCl, 20% PEG8000 (w/v), 100μM ABA, 100μM JA, 100μM SA, 5μM IAA and 5μM BAP solutions for different stress and hormone treatments. A low temperature treatment was carried out at 4°C. Treated seedlings were harvested at different time intervals (Control, 1 hr, 6 hr, 12 hr, 24 hr) and immediately frozen in liquid nitrogen for further use. Control seedlings were exposed to deionized water. For tissue specific expression study; root, stem, leaf and flower tissues were separately harvested from 120 day-old plants. Planting, treatments and harvesting were repeated three times independently for each biological replicates.

### RNA isolation and expression analysis

Total RNA was extracted from harvested seedling samples using TRI reagent (Sigma, St Louis, MO, USA). RNA was quantified by NanoDrop1000 (Thermo Fisher Scientific MA, USA). RNA-integrity was checked by separating in 1.5% denaturing agarose gel. First strand cDNAs were synthesized using Transcriptor High Fidelity cDNA synthesis kit (Roche diagnostics GmbH, Germany) according to manufacturer’s instructions. Primers for quantitative real-time PCR (qRT-PCR) analysis were designed using the primer3 v.0.4.0 software [39] from unique regions. Amplicon lengths were kept 50 to 150 bases for optimal PCR efficiency. qRT-PCR experiments were performed using three technical and three biological replicates in the Vii A 7 Real-Time PCR System (Applied Biosystems CA, USA). Each qRT-PCR reaction was performed in 10μl reaction volume containing appropriate diluted cDNA as template, 225nM of each forward and reverse primer, and 2 X Power SYBR Green PCR master mix (Applied Biosystems CA, USA). Thermal cycling conditions were 95°C for 2min followed by 40 cycles of 95°C for 15sec, 60°C for 1min. One cycle for melt curve included in the last cycle of the program. Cycling condition for melt curve was 95°C for 15sec, 60°C for 1min and 95°C for 15sec. Specificity of each pair of primers was checked through regular PCR followed by 3% agarose gel electrophoresis, and also by melting curve examination. *Elongation factor 1-α* (*EF-1α*) gene was used as internal control to normalize the variation in amount of cDNA template. Relative expressions of genes were calculated according to delta-delta Ct method of the system. The primers used in the study are listed in S2 Table. PLACE [40] search tool was used to identify *cis*-acting elements in the promoter regions of *CaCBL* genes. The enriched GO terms were predicted using the Blast2GO pro [41] tool.

## Results and Discussions

### Identification of CBL gene family in four leguminous species

*Arabidopsis* and rice each possesses ten CBL genes (*AtCBL*s and *OsCBL*s, respectively). Genes encoding CBL peptides in three legumes, *Medicago*, soybean and common bean, were identified by searching annotated gene models and EST sequences in Phytozome and NCBI databases using keyword ‘calcineurin B’ and *Arabidopsis* and rice CBL peptide sequences as queries as mentioned in methods. Search in NCBI and Phytozome databases retrieved 15 genes from soybean, 12 genes from *Medicago* and 10 genes from common bean that were predicted to encode CBL peptides. Names of these genes were kept as per their Phytozome annotation. The identified peptides were verified for signature sequences using Pfam database [42]. To identify *CBL* genes in chickpea, *Arabidopsis* and rice CBL peptide sequences were used as queries to search for orthologous peptide sequences in the recently sequenced two chickpea genome databases (kabuli and desi genotypes) [29, 30] using BLASTP. BLASTP analysis identified 9 chickpea peptide models with CBL signature sequences in kabuli draft genome database and 7 in desi draft genome. The peptide models were named as *CaCBL*s (*C*. *arietinum* CBL) and numbered according to their sequence similarity with the corresponding *Arabidopsis* CBLs. Ortholog of *Arabidopsis* CBL7 (AtCBL7) was not available in either of the chickpea genome databases. Searching both the draft genome sequences using coding sequence of *AtCBL7* as query also did not result in any orthologous sequence. Coding sequences (CDS) of all these peptides were retrieved from both the genome databases. Orthologs of AtCBL1 and AtCBL8 were not available in annotated peptide sequences of desi chickpea genome assembly. Searching desi chickpea genome assembly with kabuli *CaCBL1* and *CaCBL8* gene sequences as queries resulted in identification of genomic DNA fragments with 100% sequence identity with the query, implying that *CaCBL1* and *CaCBL8* gene sequences were present in the desi chickpea genome assembly, however, were not annotated as protein-coding genes. Gene sequences of all the corresponding CBLs from kabuli and desi chickpea showed 100% identity indicating a high sequence conservation of this gene family within cultivated chickpea varieties. Predicted genes including their 200 bp upstream and downstream sequences were reannotated individually using annotation tool Augustus [43]. cDNAs of all the *CaCBL*s were amplified from a desi (ICC4958) and a kabuli (ICCV2) genotype chickpea and subsequently sequenced. No splice variant was observed. Expectedly, the coding sequences of the corresponding *CaCBL* genes from two chickpea genotypes showed 100% identity. However, all the *CaCBL* cDNA sequences differed from the CDS predicted in the genome annotation of either one or both the chickpea types. Statistics of *CaCBL* coding sequences obtained from sequencing of their cDNA clones and their deviation from the genome annotation data were shown in Table 1. Sequences of *CaCBL* genes, corrected CDS and peptide sequences were given in S1 text and the predicted physical properties of the peptides were listed in S3 Table. Genomic locations of the *CaCBL* genes were physically mapped on kabuli chickpea genome assembly because of larger size of its pseudomolecules (S1 Fig). *CaCBL* genes were non-randomly distributed over 5 out of 8 linkage groups (LGs) of chickpea. No CBL member was present on LG 2, -3 and -8. Three *CBL*s were found on LG 5, followed by two each on LG 6 and -7, whereas LGs 1 and -4 possessed one each. Genomic distribution data of *AtCBL*s showed similar uneven distribution pattern on *Arabidopsis* genome. No *AtCBL* was present on chromosomes 2 and -3 whereas, 6 out of 10 members were located on chromosome 4. Out of nine, seven *CaCBL* genes were in positive orientation as opposed to 6 negatively oriented *AtCBL* genes. Sequences of all the CBL peptides mentioned above including the CBL peptides from *Arabidopsis* were aligned for sequence comparison (S2 Fig).

**Table 1:** Characteristics and chromosomal locations of chickpea *CBL* genes.

Gene	Linkage group	Position in Chromosome (base)	Gene length (bp)	CDS length (bp)	Peptide length (aa)	Desi Annotation CDS length (bp)	Kabuli Annotation CDS length (bp)	Exons	Introns
CaCBL1	7	10405274–10407956	2682	642	213	-	642	8	7
CaCBL2	5	15443–18352	2909	627	208	627	630	7	6
CaCBL3	5	23684107–23687221	3114	681	226	621	621	8	7
CaCBL4	5	45368828–45366849	1979	669	222	609	669	8	7
CaCBL5	6	4624193–4627270	3077	645	214	576	576	8	7
CaCBL6	1	6621696–6624338	2642	681	226	1233	1266	8	7
CaCBL8	6	4621130–4623438	2308	648	215	-	567	8	7
CaCBL9	7	31718042–31722274	4232	777	258	480	777	9	8
CaCBL10	4	45144713–45139122	5591	759	252	759	696	9	8

### Sequence comparison of legume CBL peptides

Existence of CBL-encoding genes in the genomes of unicellular plant species indicates conservation of Ca^2+^-signaling system in plant kingdom. The smallest free-living eukaryote *Ostreococcus* and unicellular green algae *Chlorella* possess one CBL-encoding gene each, while the moss *Physcomitrella* possesses five genes that show similarity with CBL-encoding genes. The number of CBL-encoding genes increased with evolution of plant lineage suggesting increased complexity in Ca^2+^-signaling system. In general, the CBL peptides are composed of 200 to 260 amino acids. However, one gene each from *Medicago*, common bean and *Physcomitrella* (Medtr3g060730.1, Phvul002G002300.1, Ppls65_59v6.1, respectively) encode peptides of less than 200 amino acid-length and two genes from *Medicago* (MedtrAC235758_37.1 and Medtr4g113510.1) encode peptides comprising more than 300 amino acids. CBL peptides are known to have two pairs of EF-hand motif to bind a maximum of four calcium ions. However, according to Pfam analyses, smaller CBL peptides encoded by Phvul002G002300.1 and Ppls65_59v6.1 of common bean and *Physcomitrella*, respectively, possess only one pair of EF-hands and, therefore, do not qualify as potential CBL proteins. Sequence alignment shows that variations among the CBL sequences arise mostly due to variation in N-terminal sequences, while the EF-hand region and the C-termini are conserved. The longer proteins encoded by MedtrAC235758_37.1 and Medtr4g113510.1 are due to extra long C-terminus and N-terminus, respectively. The extra long N-terminus of Medtr4g113510.1 was due to ten repeats of ‘QFLH(H/S)RLGSRR’ motif, which may have arisen due to recombination error.

The only calcineurin B gene of yeast (YKL190W) encodes a 175 aa-long peptide. The peptide possesses the conserved MGXXXS/T motif for N-myristoylation. *Arabidopsis* CBL proteins (AtCBL1,-4,-5,-9) also possessed a conserved cysteine residue followed by a glycine residue within the N-myristoylation site for S-acylation suggesting dual modification. These modifications are required to target the proteins to membrane and ultimately for their activity [23, 30, 31, 44]. Three CBL peptides each from *Medicago* (Medtr5g13560.1, Medtr3g091440.3, MedtrAC235758_37.1), common bean (Phvul03g225900.1, Phvul09g052700.4, Phvul02g299300.1) and chickpea (*CaCBL*1,-4,-5) and seven CBL peptides from soybean (Glyma05g05580.1, Glyma11g04160.2, Glyma17g15893.2, Glyma06g13420.1, Glyma04g41430.2, Glyma05g36800.2, Glyma08g02740.1) possess these conserved motifs and are predicted to be located in plasma membrane. AtCBL2, -3 and -6 are localized in tonoplast [44]. They possess MXQCV/I/L motif at their N-terminus. Similarly *CaCBL*2, -3 and -6 also possess similar motif at their N-terminus and so are predicted to be localized at tonoplast.

### Evolutionary and phylogenetic analyses of legume CBL family

Soybean and *Medicago* possess more CBL-encoding genes than *Arabidopsis* and rice. However, it is to be noted that soybean has undergone a recent whole genome duplication (WGD) approximately 13 million years ago (mya) resulting in highly duplicated genes [45]; and a high gene count in *Medicago* is a resultant of extensive genome-wide local gene duplication [46]. Six pairs of soybean CBL genes (Glyma17g15893.2 and Glyma05g005580.1; Glyma05g36800.2 and Glyma08g02740.1; Glyma04g41430.2 and Glyma06g13420.1; Glyma07g39936.1 and Glyma17g00830.3; Glyma07g01300.2 and Glyma08g20700.2; Glyma18g08230.2 and Glyma08g44580.1) are located in the paralogous blocks in the genome and according to the rate of synonymous substitution (Ks) these pairs were diverged by 8-13my, which agrees with proposed period of WGD in soybean. Therefore, it appears that the protosoybean before WGD possessed 9 CBl-encoding genes. Four CBL-encoding genes (Medtr2g027440.1; Medtr2g027480.1, Medtr2g027500.1; Medtr2g027520.1) of *Medicago* are placed tandemly on chromosome 2. Comparison of pair-wise synonymous substitution rates among all the combinations suggested that Medtr2g027440.1 and Medtr2g027520.1 were separated by about 23 mya, after *Medicago* diverged from chickpea about 25–30 mya [30]. Similarly, Medtr2g027500.1 and Medtr2g027520.1 were separated by about 19 mya. Origin of Medtr2g027440.1 or Medtr2g027480.1 is very recent as they are separated only by 8 thousand years according to Ks value. Hence, if one gene is considered as the origin of other three genes in this cluster, *Medicago* also encode total nine original CBL genes. It has already been mentioned that the peptide encoded by Phvul002G002300.1 of common bean is very small and does not qualify to be a potential CBL protein. All the gene analyses described above strongly suggest that the legume genomes or at least genome assemblies of these four leguminous species encode nine original CBL-encoding genes. Any excess in CBL-encoding peptides was resulted due to whole genome duplication or genome-wide local duplication in soybean and *Medicago* after chickpea diverged from *Medicago*. Similarly, higher number of CBL in *Arabidopsis* might have resulted from its WGD about 65–72 mya [47] as two pairs of its CBL genes (AtCBL1 and AtCBL9; AtCBL2 and AtCBL3) located on chromosomes 4 and 5 are paralogous to each other and were separated during WGD mentioned above.

**Fig 1:**
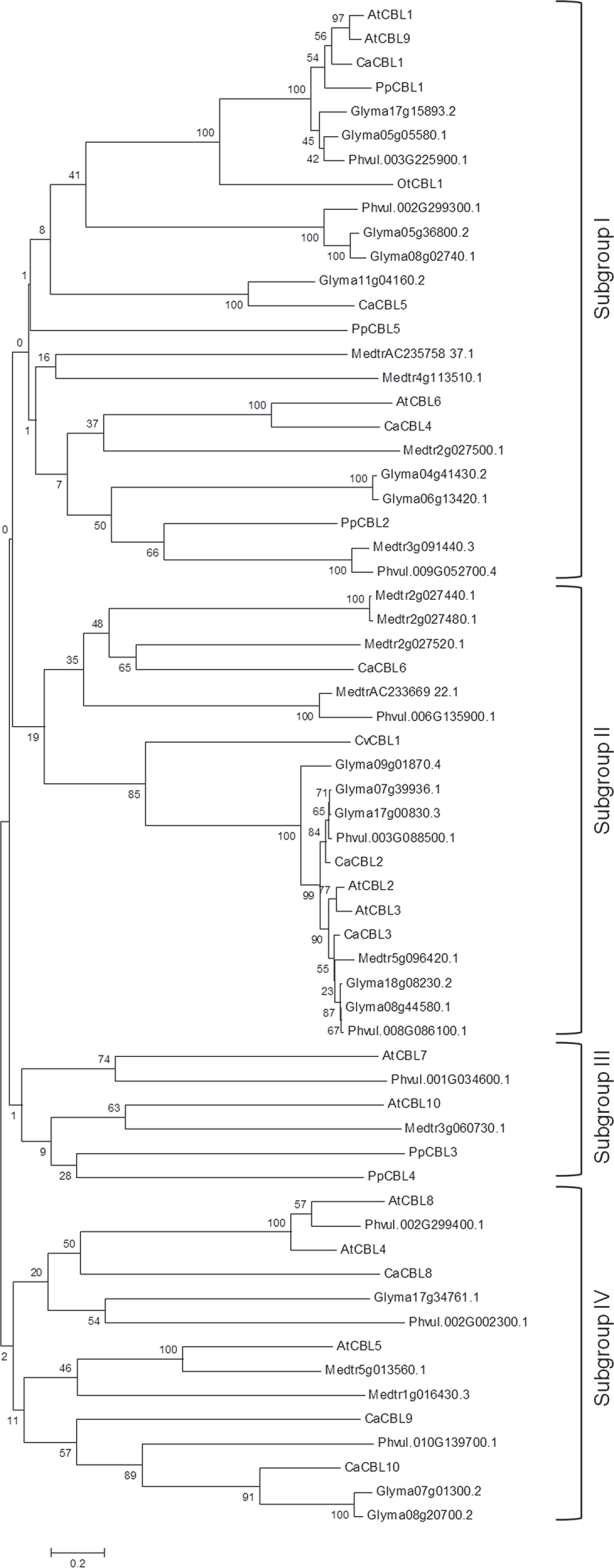
Phylogenetic relationship of CBL proteins from soybean, common bean, *Medicago truncatula* and chickpea with CBLs from other species. Peptide sequences were aligned by Clustal X and the tree was constructed by neighbour-joining method by using MEGAv6. The number denotes the bootstrap values.

To study phylogenetic relationship between CBL proteins from different species, peptide sequences of CBLs from *Arabidopsis*, chickpea, *Medicago*, common bean, soybean, *Chlorella*, *Ostreococcus* and *Physcomitrella* were used to construct an unrooted tree. Phylogenetic analysis suggested that legume CBL proteins can be distributed into 4 subgroups (Fig 1). As expected, only two legume CBLs, one each from *Medicago* and common bean, grouped in the same clade with AtCBL7 and AtCBL10. Nine chickpea CBLs were equally distributed in three other groups and distribution of other CBLs from other legume plants were almost equal in these three groups. Sequences of AtCBL1 and AtCBL9 are very similar and so the sequences of AtCBL2 and AtCBL3. They are grouped with each other with high bootstrap values indicating that they are results of gene duplication. However, their orthologs in chickpea are grouped separately suggesting that they were originated through a different course of evolution. The duplicated genes of soybean and *Medicago* were also grouped together.

### Evolutionary analyses of gene and protein structures of CBL family

**Fig 2:**
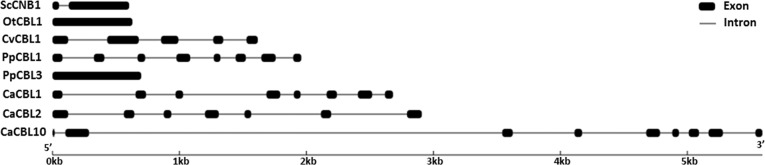
The exon/Intron structure of representative CBL genes from various plant species. Introns and exons are represented by lines and black boxes, respectively. The length of lines and boxes correspond to intron-exon size accordingly. The exon-intron structures of *CaCBL* genes were determined by aligning their cDNA sequences to the corresponding gene sequences using Gene Structure Display Server 2.0 program. Exon/Intron structures of all the PpCBL and *CaCBL* genes are shown in S3 Fig.

Comparison of gene and peptide sequences of CBL gene family reveals an evolution of gene structure. 604 base pair (bp)-long calcineurin B gene of yeast encodes a 175 aa-long peptide derived from two coding regions and the protein possesses a motif for N-myristoylation. The only CBL gene of *Ostreococcus* (Ot16g00900, *OtCBL1*) is of simple structure without any intron (Fig 2 and S3 Fig) and the protein is devoid of conserved motif for N-myristoylation (S2 Fig). OtCBL1 peptide sequence shows only about 30% amino acid identity with the yeast calcineurin B, indicating that the OtCBL1 might not have been derived from yeast calcineurin B or it has lost myristoylation motif and intron in the evolution. The *Chlorella* CBL gene (FJ901249.1, CvCBL1) is longer (1617 bp) than those encoding yeast calcineurin B (604 bp) and OtCBL1 (630 bp) and its protein coding sequence (CDS) is distributed in five coding regions showing appearance of complex gene structure with evolution. Increase in intron number is seen as an increase in the probability of differential splicing and ability to absorb mutations. The *Chlorella* CBL possesses motif for myristoylation. *Physcomitrella CBL* genes could be divided into two groups according to their gene structure. PpCBL2 (Pp1s4_347V6.1) and PpCBL3 (Pp1s107_63V6.1) are encoded by single coding regions of their ~650 bp-long genes, while *PpCBL1* (Pp1s28_66V6.2) and *PpCBL4* (Pp1s64_196V6.1) are longer (1960 bp and 2268 bp) in size and their coding sequences are distributed in eight coding regions. However, both *PpCBL1* and *PpCBL2* encode peptides of same length (213 aa) and possess MGXXXS motif, whereas PpCBL3 and PpCBL4 do not have this motif. Therefore, *Physcomitrella* possesses CBL genes with both simple and complex gene structures and its CBL genes encode proteins with and without myristoylation motif. Multiexonic *CvCBL1* gene shows initiation of complex gene structure in unicellular algae. It appears that CBL proteins with or without N-myristoylation motif have different origins. Notably, CBL proteins appear to be absent in algae *Volvox carteri* and *Chlamydomonas rheinhardtii*, which are phylogenetically placed between *Chlorella* and *Physcomitrella* suggesting different calcium-signaling mechanism in these species. Re-appearance of CBL gene family in moss *Physcomitrella* with both mono- and multi-exonic genes and proteins with or without N-myristoylation motif indicates a convergence of both the types of CBL genes in this species.

This complex CBL gene structure with multiple coding regions (varies from seven to nine) is maintained throughout the higher plants. Seven (*CaCBL1*-*CaCBL8*) out of nine *CaCBL* genes are 2–3 kb long, whereas *CaCBL9* and *CaCBL10* genes are longer (4–5 kb). However, they encode peptides of similar sizes (21–28 kDa). Interestingly, irrespective of much larger sizes of *CaCBL9* and *CaCBL10* genes, number of coding regions did not change. Only these two *CaCBL* genes possess nine coding regions in contrast to *Arabidopsis*, while only *AtCBL10* possesses nine coding regions.

**Fig 3:**
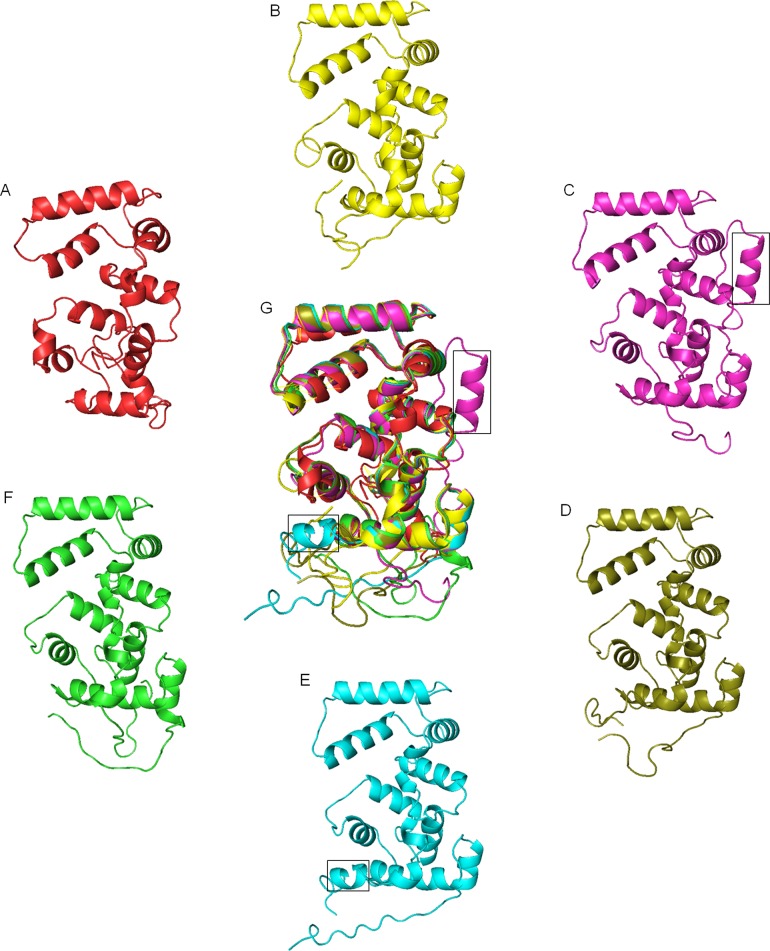
Comparison of structures of CBL proteins from different species. Protein structure of calcineurin B of *Saccharomyces cerevisiae* (A) and CBL1 from, *Ostreococcus tauri* (B), *Chlorella variabilis* (C), *Physcomitrella patens* (D), chickpea (E) and *Arabidopsis* (F) were generated using Phyre2 remote homology modeling server. All five structures from different species were analysed by pyMOL program. All the structures were superimposed (G) to show presence of extra helix (in boxes) in CBL proteins of *Chlorella* and chickpea.

Crystal structure of AtCBL2 [48] was used to derive peptide structures of OtCBL1, CvCBL1, PpCBL1, AtCBL1 and *CaCBL*1. Although these peptide sequences share 38–81% amino acid identity, they maintain very similar protein structure (Fig 3), suggesting a high conservation of function. Structure of yeast calcineurin B is also presented to show an overall similarity in structure. Structure of CvCBL1 showed some deviation from those of OtCBL1 and other plant CBLs presented in the figure. All the CBL peptides are devoid of beta sheets and composed of helix and loops. Yeast calcineurin B protein and OtCBL1 have 11 helices. All other CBLs in higher organisms are composed of 13–14 helices. The last two helices were broken into four helices in CBL peptides of higher organisms to result in 13 helices. CvCBL1 possesses 14 helices because it has a long N-terminus and the sequence ‘VEVEAKA’ in the N-terminal domain forms an extra helix. *CaCBL*1, with a small N-terminus, is composed of 14 helices because of a small C-terminal helix composed of a sequence stretch ‘FVFNS’. Although, this sequence stretch is fairly conserved in other CBLs (S2 Fig), it formed loops in those peptides, but a helix in *CaCBL*1. In the merged peptide structure these two deviations in *CaCBL*1 and CvCBL1 could be identified.

### The expression pattern of *CaCBL* genes in different tissues

**Fig 4:**
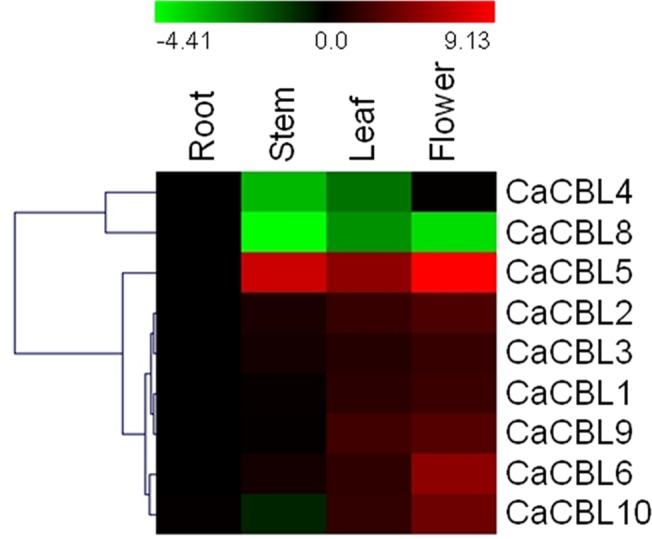
Tissue specific gene expression analysis of chickpea *CBL* genes. For tissue specific expression studies, total RNA was isolated from different tissues of chickpea plants. cDNA were prepared and expression pattern of all chickpea *CBL* genes were analysed by qRT-PCR and presented as heatmap. Chickpea *elongation factor 1-α (EF-1α)* mRNA was used as internal control for normalization. The scale bar represents relative expression values. Hierarchical clustering has been shown at left. Relative fold expression values are presented as bar diagram in S4 Fig.

The expression pattern of the genes can provide important information for understanding their function. Expression pattern of *CaCBL* genes in different tissues were studied by qRT-PCR. The mRNA accumulation of each gene in root, stem, leaf and flower tissues was assessed. Except *CaCBL8*, other *CaCBL* genes are expressed at a higher level in flower in comparison to the other tissues, indicating important role of CBL-CIPK pathway in reproductive development (Fig 4). Specifically, expression ratio of *CaCBL*5 in flower to other tissues is exceptionally high. *AtCBL1* and *AtCBL9* were reported to function in pollen germination and pollen tube growth [49]. *CaCBL4* is expressed more in root and flower. *CaCBL8* was mostly expressed in roots. Expressions of *CaCBL1*, *-2*, *-3*, *-5* and *-6* in root were lowest among all the tissues. Although, gene and peptide structures of CBL family in higher plants are highly conserved, their expression patterns vary from plant to plant. *Arabidopsis* and *B*. *napus* are phylogenetically very close. Inspite of that their CBL genes showed entirely different patterns of expression. Likewise, tissue-specific expression pattern of *CaCBL* genes do not follow the pattern of those from *Arabidopsis*. However, higher expression of *AtCBL1*, *-2* and *-3* in flower corresponded with similar expression of their orthologs in chickpea [18, 50].

### Expression profile of *CaCBL* genes under abiotic stresses

**Fig 5:**
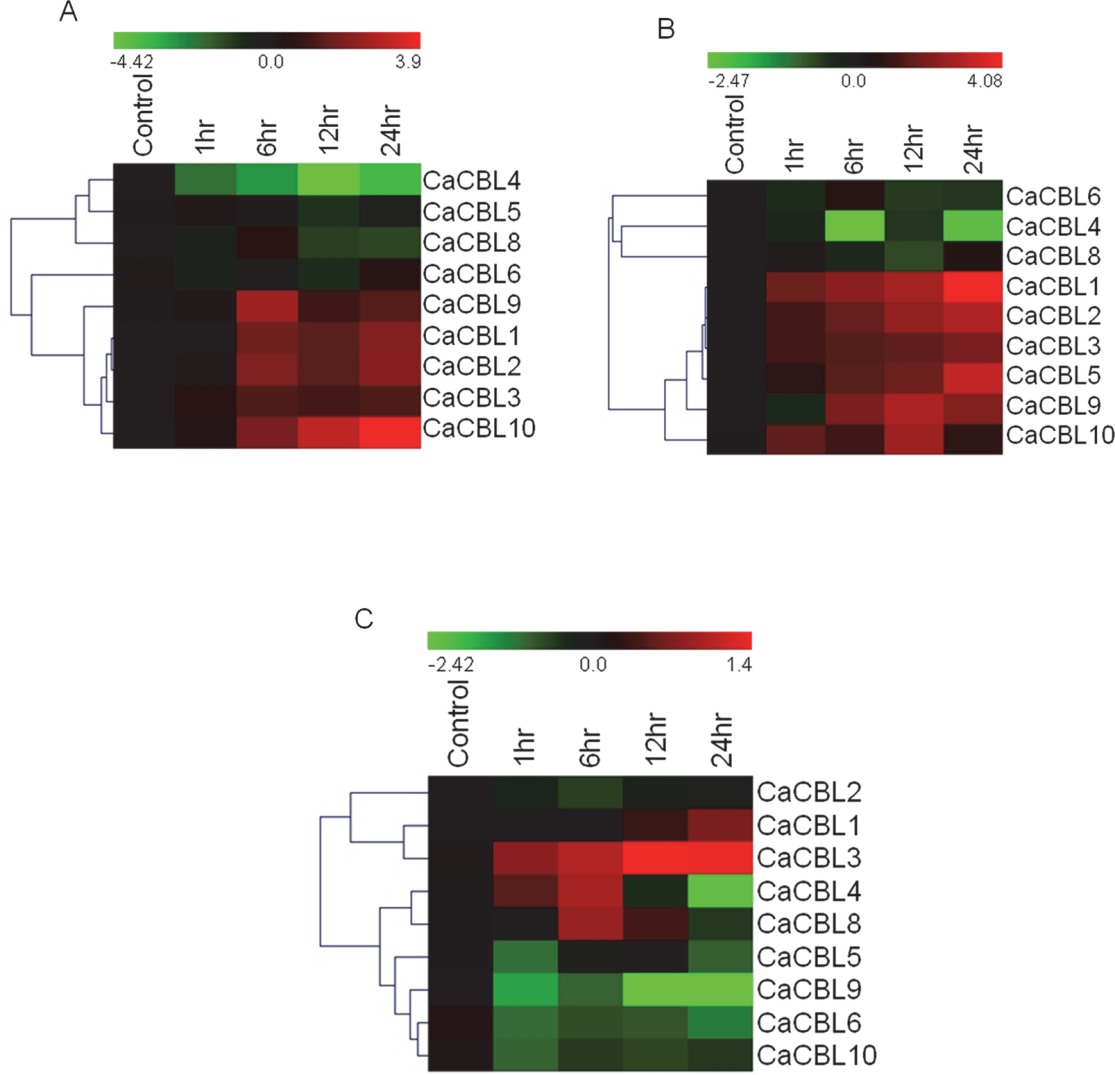
Relative transcript levels of chickpea *CBL* genes in response to different abiotic stress treatments. 6 day-old chickpea seedlings were exposed to 250 mM NaCl (A), 20% PEG (B) and Cold at 4°C (C) and samples were harvested at different time interval as mentioned for relative expression studies by qRT-PCR and presented as heatmap. The scale bar represents relative expression values. Hierarchical clustering has been represented at left. Relative fold expression values are presented as bar diagram in S4 Fig.

The first CBL gene (AtCBL4/SOS3) was identified in the screening for salt-overly-sensitive phenotype. Subsequent reports suggest that most of the CBL and CIPK genes were found associated with signaling related to abiotic stress and abiotic stress-related hormones. To investigate the potential role of *CaCBL* genes in abiotic stress response, expression studies were performed under different conditions of stresses for different time periods. 6 day-old chickpea seedlings were treated with 250mM NaCl (salt) or 20% PEG (dehydration) or 4°C (cold). The relative expressions were converted to log 2 values of fold changes and presented as heatmap with reference to the value at control condition. Exposure to 250mM sodium chloride appears to have insignificant effect on expression of *CaCBL5*, *-8* and *-6* (Fig 5A). Surprisingly, expression of *CaCBL4* was downregulated by salt exposure, which suggested that it functions differently from its ortholog in *Arabidopsis*. Expression of other *CaCBL*s was increased by salt treatment. Several studies have suggested that overlapping signaling pathways operate in response to drought and salinity. Apart from *CaCBL5*, expression of all other chickpea CBL genes under PEG treatment followed same pattern of their expression in response to salinity. Expressions of *CaCBL6*, *-4* and *-8* were either downregulated or unaltered under PEG treatment. In contrast to salt treatment, dehydration enhanced expression of *CaCBL5* by ten fold. *CaCBL10* registered the highest fold (16-fold) of expression under salinity, while its expression under dehydration was periodically upregulated only at 1hr and 12hr. *CaCBL1* registered the highest fold (17-fold) of expression under dehydration at 24hr (Fig 5B).

Calcium plays an important role in the CBF-mediated cold response pathway by induction of some cold-inducible genes like COR6 and KIN1 genes of *Arabidopsis* [1, 51, 52]. A previous report suggested that the expression of *AtCBL1* was induced by cold stress and had a role in regulating cold response genes [53]. *Arabidopsis* mutant with compromised expression of CIPK6 showed decreased expression of DREB1A [54]. Expression patterns of *CaCBL* genes under cold treatment were totally different from their expression patterns under salt and dehydration treatment suggesting distinct calcium signaling in cold stress. Unlike enhanced expression under salt and dehydration treatment exposure to low temperature resulted in decreased expression of most of the *CaCBL*s. Only *CaCBL3* expression was consistently but not significantly (2.5-fold at 24hr) increased by cold treatment. Expression of *CaCBL4* was increased up to 6hrs (2-fold) (Fig 5C). Expression range of *CaCBL* genes under low temperature treatment was also much lower than those with salt and dehydration treatments.

### Expression profile of *CaCBL* genes in response to phytohormones

Phytohormones integrate growth and development and long-term response to external environmental inputs. Abscisic acid (ABA) is known to increase cytosolic Ca^2+^ level [55]. Previous studies with *Arabidopsis* suggested AtCBL1 and AtCBL9 both form a complex with AtCIPK1 separately and play important roles in ABA-mediated stress responses [13]. We analysed expression profiles of *CaCBL*s in response to different phytohormones to study their involvement in hormone-mediated signaling. 6 day-old chickpea seedlings were treated with major phytohormones, such as 100μM ABA, 5μM IAA (Indole acetic acid, auxin), 5μM BAP (Benzylaminopurine, cytokinin), 100μM MeJ (Methyl jasmonate) and 100μM SA (Salicylic acid).

**Fig 6:**
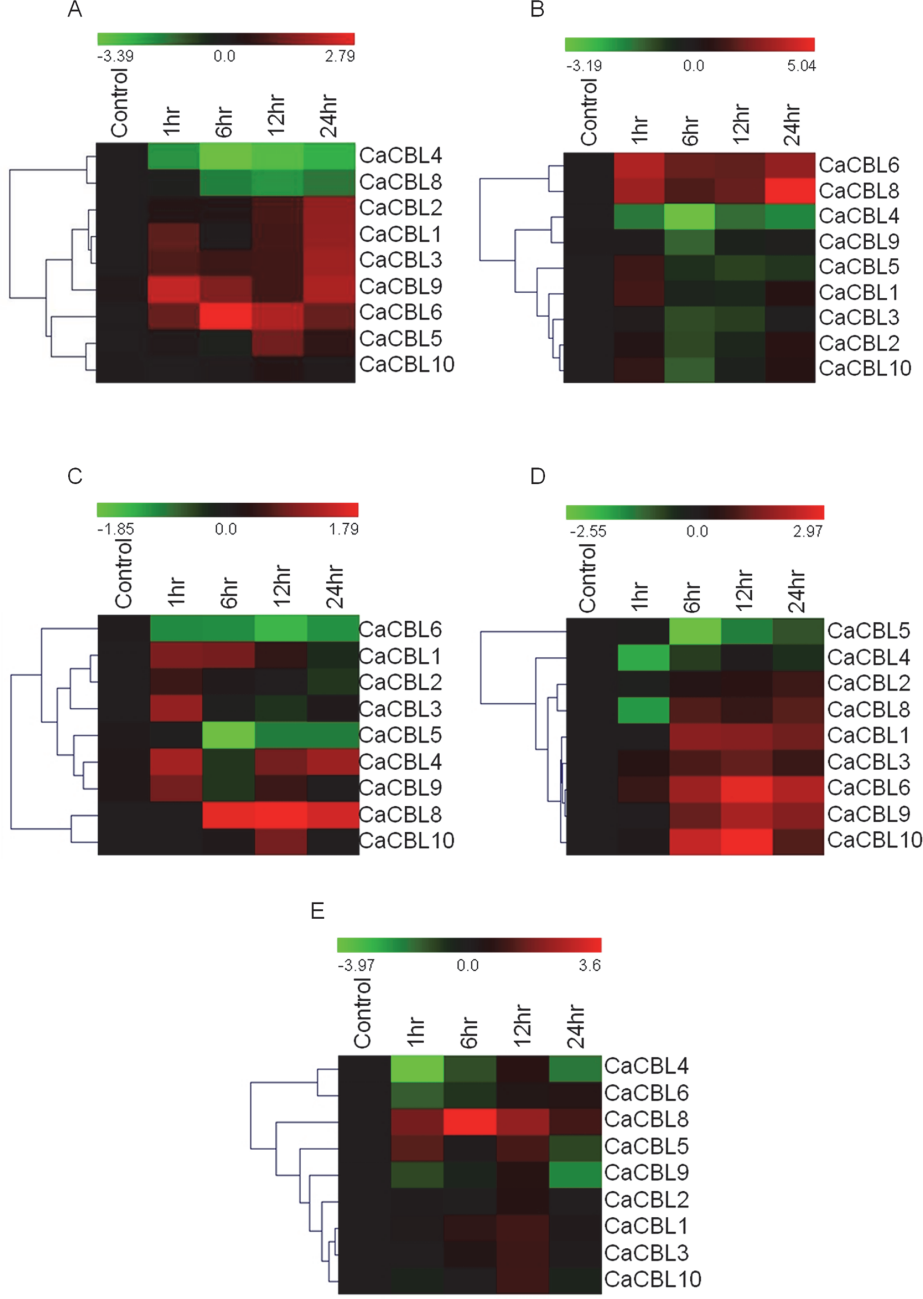
Relative transcript levels of chickpea *CBL* genes in response to treatment with various phytohormones. Expression profiles of *CBL* genes in chickpea seedlings exposed to Abscisic acid (A), BAP (B), IAA (C), Salicylic acid (D) and Methyl Jasmonate (E) for different period by qRT-PCR and presented as heatmap. The scale bar represents relative expression value. Hierarchical clustering was played in data analysis. Relative fold expression values are presented as bar diagram in S4 Fig.

Expressions of *CaCBL4* and *-8* are consistently decreased with time in response to ABA treatment. *CaCBL10* expression did not alter by the treatment. Expressions of rest of the *CaCBL* genes were enhanced with exposure period, however, not steadily. The maximum fold increase was observed for *CaCBL6* gene expression, which was enhanced by more than 6-folds after six hours of treatment (Fig 6A). *CaCBL1*, *-2*, *-3* and *-9* registered similar enhancement in expression. Expression patterns of *CaCBL1* and *-9* in response to ABA are in accordance with the role of their orthologs in *Arabidopsis* in ABA-mediated signaling.

Auxins and cytokinins are key growth hormones that simulate all important developmental processes like cell division, cell elongation, root initiation and bud formation. They work synergistically as well as antagonistically depending on context. AtCBL1 and 9 functions in pollen germination and pollen tube growth [49]. AtCBL2 and AtCBL3 affect seed size and embryonic development [56]. To investigate possible role of *CaCBL*s in auxin and cytokinin signaling, expression level of all chickpea CBLs under auxin (IAA) and cytokinin (BAP) treatment was examined (Fig 6B and 6C). *CaCBL6* and *CaCBL4* registered entirely opposite expression profiles under auxin and cytokinin treatments. *CaCBL6* transcripts level increased steadily with cytokinin treatment, however, showed steady decrease under auxin treatment. In contrast, *CaCBL4* expression enhanced with auxin treatment except after 6 hr time point, while it showed steady decrease under cytokinin treatment. Interestingly, *CaCBL8* expression levels increased under both auxin and cytokinin treatments. Expression range of *CaCBL* genes under auxin treatment is the narrowest among the expression ranges under different hormone treatments, while cytokinin treatment caused highest change in fold expressions of *CaCBL* genes. Therefore, enhanced expression of *CaCBL8* (30-folds) under BAP treatment appears to be more significant than its response to auxin treatment (3-folds). After BAP treatment, expressions of majority of chickpea CBLs were down regulated except *CaCBL6* and *CaCBL8*. Expressions of *CaCBL6* and *CaCBL8* were increased up to 12- and 9-folds, respectively, after 1hr and then quickly decreased. Their expression recovered again to 8- to 32-fold, respectively, at 24 hrs. *CaCBL1* and *CaCBL5* transcript levels quickly increased within 1 hr and then decreased. *CaCBL4* and *CaCBL9* expression was consistently down regulated.

Cytosolic Ca^2+^ concentration is modulated by microbe infection or by treatment with microbe-associated molecular patterns (MAMP) [57]. Salicylic acid (SA) and jasmonic acid (JA) are phytohormones related to plant defence against pathogen infection. SA boosts plant immunity by inducing expression of pathogenesis-related proteins. Along with SA involved in systemic acquired resistance (SAR). Plants with compromised expression of SA are more susceptible to pathogen infection. JA is critical for plant defence against herbivory and necrotrophic pathogens. RNAi-mediated suppression of *OsCIPK14* and *OsCIPK15* expression suppressed MAMP-mediated reactive oxygen species production in rice [58]. Recently a report on tomato CBL10 established a mechanistic link between Ca^2+^ and ROS signaling in plant immunity [27]. These reports provided a new dimension to CBL-CIPK pathway and its role in plant defence should be further investigated. Expression levels of all chickpea CBLs were examined in response to SA and methyl jasmonate (MeJ), a derivative of JA, treatment to investigate their involvement in SA and JA signaling. Expression of seven out of nine *CaCBL*s were up regulated in response to SA treatment (Fig 6D). The transcript levels of six chickpea CBL genes (*CaCBL1*, *CaCBL3*, *CaCBL6*, *CaCBL8*, *CaCBL9* and *CaCBL10*) were significantly increased in response to SA from 6 hr onwards. A minor alteration in *CaCBL2* expression at different time points was also observed. An 8- fold increase in *CaCBL10* transcript level was in accordance with the role of its ortholog in *Arabidopsis*. SA treatment caused a decline in the expression of *CaCBL*4 and *CaCBL*5. Apart from *CaCBL8* none of the *CaCBL* genes showed a steady expression pattern upon MeJ treatment. *CaCBL8* registered a steady increase in expression with a 12-fold of enhancement at 6 hrs. In contrast, *CaCBL4* transcript level declined except at 12 hr time point. *CaCBL1* and *CaCBL3* expressions increased marginally up to 12hr (2-fold). Other *CaCBL* genes did not show significant alteration upon MeJ treatment (Fig 6E). As mentioned above, expression patterns of *CaCBL* genes under different stresses and phytohormones did not follow of those of their orthologs in *Arabidopsis*. However, enhanced expression of AtCBL1 and -9 in response to ABA and salt treatments, and higher expression of AtCBL3 in response to cold treatment corresponded with expression patterns of orthogous chickpea genes. Quantitative values with standard deviations among three biological replicates for all the expression analyses have been presented as bar diagrams in S4 Fig.

Chickpea *CBL* genes showed diverse expression patterns in response to abiotic stress and hormone treatments (S4A Table). Enhanced expression of *CaCBL1*, *-2*, *-9* and *-10* in response to salt, dehydration and ABA could be correlated with presence of well-known *cis*-acting elements related to these stresses such as, ACGTATERD1[59], ABRELATERD1[59], CBFHV (DRE) [60] and DRECRTCOREAT [61] in the promoter sequences of these genes (S4B Table). Increased expression of *CaCBL6* and *-8* in response to both defence and development related hormones could be correlated with presence of *cis*-acting elements eg. ARR1AT [62], ERELEE4 [63], WRKY71OS [64] and GT1CONSENSUS [65] known to be responsive to development and defence. Interestingly, POLLEN1LELAT52 [66] is present in promoter sequences of all the genes correlating their strong expression in reproductive organs. *CaCBL4* and *-8* showed higher expression in root. While both the genes exhibited downregulation in response to abiotic stresses and related hormone treatment, *CaCBL8* expression was upregulated in response to development and defence related hormones indicating its involvement in these processes. Expression pattern of CaCBL4 is surprising as its expression decreased or unaltered in response to all the treatments except MeJ. We also correlated the expression profiles of *CaCBL* genes with GO annotation (S5 Fig). Molecular function analysis suggested that all *CaCBL* genes have calcium binding domains and they are involved in various biological functions, primarily in calcium mediated signaling, multidimensional cell growth, regulation of ion homeostasis, stress responses and related organelle functions.

## Conclusion

Our present study identified nine CBL genes in chickpea genome and suggested that more number of CBL genes in other legumes is result of gene duplication. This is not unusual because grapevine (8 VvCBLs) and sorghum (6 SbCBLs) have less number of CBL genes [21]. It is possible that more *CaCBL* genes might be identified once the advanced genome sequence is available. However, as *Medicago*, soybean and common bean possess original basic set of nine functional CBLs, it is unlikely that chickpea would encode any more CBL genes as it did not undergo any recent whole genome duplication or local gene duplication. The predicted peptide and coding sequences were corrected by cloning individual cDNAs followed by sequencing. No ortholog of *AtCBL7* gene was identified in chickpea. *CaCBL* genes, like several *AtCBL* genes, showed an overall higher expression in flower, suggesting importance of Ca^+2^-signaling and specifically CBL-CIPK signaling in reproductive development of plant, which could be focus in near future. Most of the *CaCBL* genes showed enhanced expression under salt and PEG treatments corresponding with widely published importance of this pathway in abiotic stresses. We tried to compare the 5’-upstream sequences of *CaCBL* genes with those of *AtCBL* genes to search for common *cis*-acting elements, but arrived at a conclusion that they are diverse and no significant similarity had been observed between the orthologs in other plants. The diverse expression patterns are expected for diverse developmental queues, and biotic and abiotic stresses faced by a plant. These new CBL sequences and expression information will be useful in further investigation of the function of CBL genes in chickpea.

## Supporting Information

S1 Fig Physical mapping of *CaCBL* genes on chickpea pseudomolecules. (PDF)

S2 Fig Multiple sequence alignment of *CBL* genes from various plant species. (PDF)

S3 Fig Exon/Intron structure of *CBLs* and *calcineurin* B genes from various species (PDF)

S4 Fig (A-I) Relative expression values of *CaCBL* genes in different tissues of chickpea, and in chickpea seedlings exposed to 20% PEG, 250mM NaCl, 100μM ABA, 5μM BAP, 5μM IAA, 100μM MeJ, 100μM SA and cold (4°C) for different periods presented as bar diagram. (PDF)

S5 Fig (A, B) GO annotation of 9 *CaCBL* genes on the basis of their molecular function and biological processes. (PDF)

S1 Table *CaCBL* primers for cloning and sequence confirmation. (DOCX)

S2 Table *CaCBL* primers for qRT-PCR. (DOCX)

S3 Table Physical properties of 9 *CaCBL* peptides. (DOCX)

S4 Table (A, B) A. Qualitative expression profiling of CaCBL genes in different tissues and in response to different treatment based on qRT-PCR result. B. Identification of *cis*-acting elements in 1.2 kb promoter regions of *CaCBL* genes.
(DOCX)

S1 Text Corrected sequences of *CaCBL* genes, CDS and proteins. (DOCX)
