# Supplementary material for: Investigation of Genes Encoding Calcineurin B-Like Protein Family in Legumes and Their Expression Analyses in Chickpea (Cicer arietinum L.)
Source: PLoS One. 2015 Apr 8;10(4):e0123640. doi: 10.1371/journal.pone.0123640 (PMC4390317; doi:10.1371/journal.pone.0123640)
Supplement: S1 Fig — (PDF) [file pone.0123640.s001.pdf]

S1 Fig. Physical mapping of *CaCBL* genes on chickpea pseudomolecules

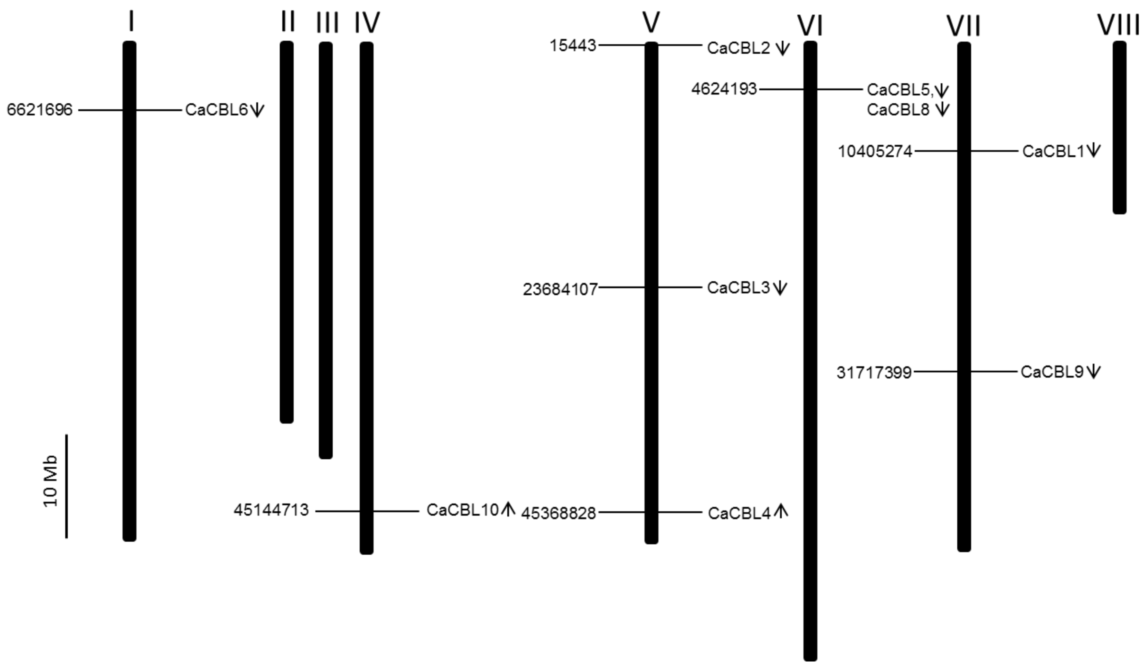

S1 Fig. Physical mapping of *CaCBL* genes on chickpea pseudomolecules.The chromosomal position of each *CaCBL* was mapped on kabuli chickpea genome assembly. The arrow next to gene id shows the direction of transcription. The scale represents a 10 Mb chromosomal distance.
