# Supplementary material for: Investigation of Genes Encoding Calcineurin B-Like Protein Family in Legumes and Their Expression Analyses in Chickpea (Cicer arietinum L.)
Source: PLoS One. 2015 Apr 8;10(4):e0123640. doi: 10.1371/journal.pone.0123640 (PMC4390317; doi:10.1371/journal.pone.0123640)
Supplement: S3 Fig — (PDF) [file pone.0123640.s003.pdf]

**S3 Fig.** Exon/Intron structure of *CBLs* and *calcineurin B* genes from various species

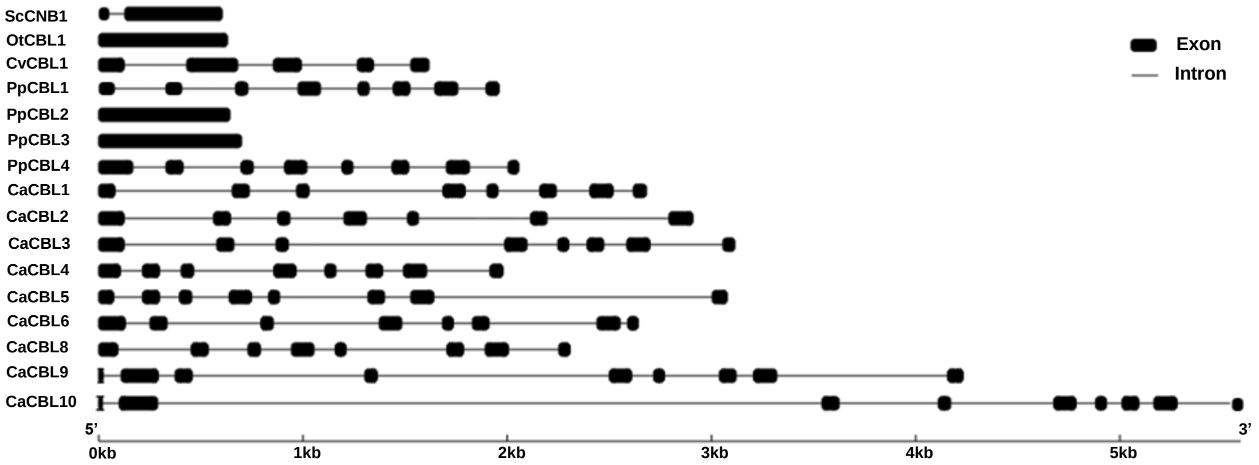


**S3 Fig.** Exon/Intron structure of *CBL* genes of *O. tauri (OtCBL1), C. variabilis (CvCBL1), P. patens (PpCBL1-4), C. arietinum (CaCBL1-10)* and *calcineurin B* gene of *S. cerevisiae.* Exons and Introns are represented by black boxes and lines, respectively. Scale is represented at the bottom of figure for estimation of exon, intron and gene sizes. The figure was generated by Gene Structure Display Server 2.0.
