# Supplementary material for: Investigation of Genes Encoding Calcineurin B-Like Protein Family in Legumes and Their Expression Analyses in Chickpea (Cicer arietinum L.)
Source: PLoS One. 2015 Apr 8;10(4):e0123640. doi: 10.1371/journal.pone.0123640 (PMC4390317; doi:10.1371/journal.pone.0123640)

S4 (A-I) Fig. Relative expression values of *CaCBL* genes in chickpea

S4A Fig. Relative expression values of *CaCBL* genes in different tissues of chickpea analysed by qRT-PCR

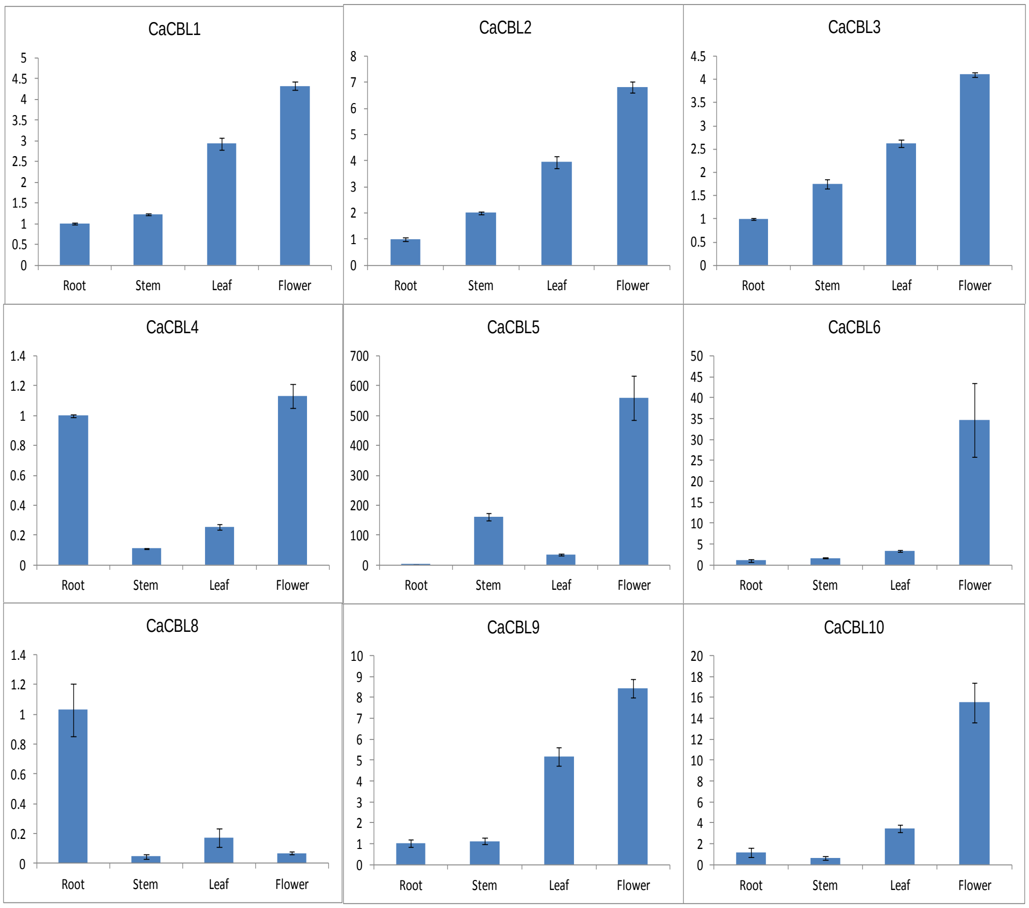

S4B Fig. Relative expression values of *CaCBL* genes in chickpea seedlings exposed to 20% PEG for different period analysed by qRT-PCR

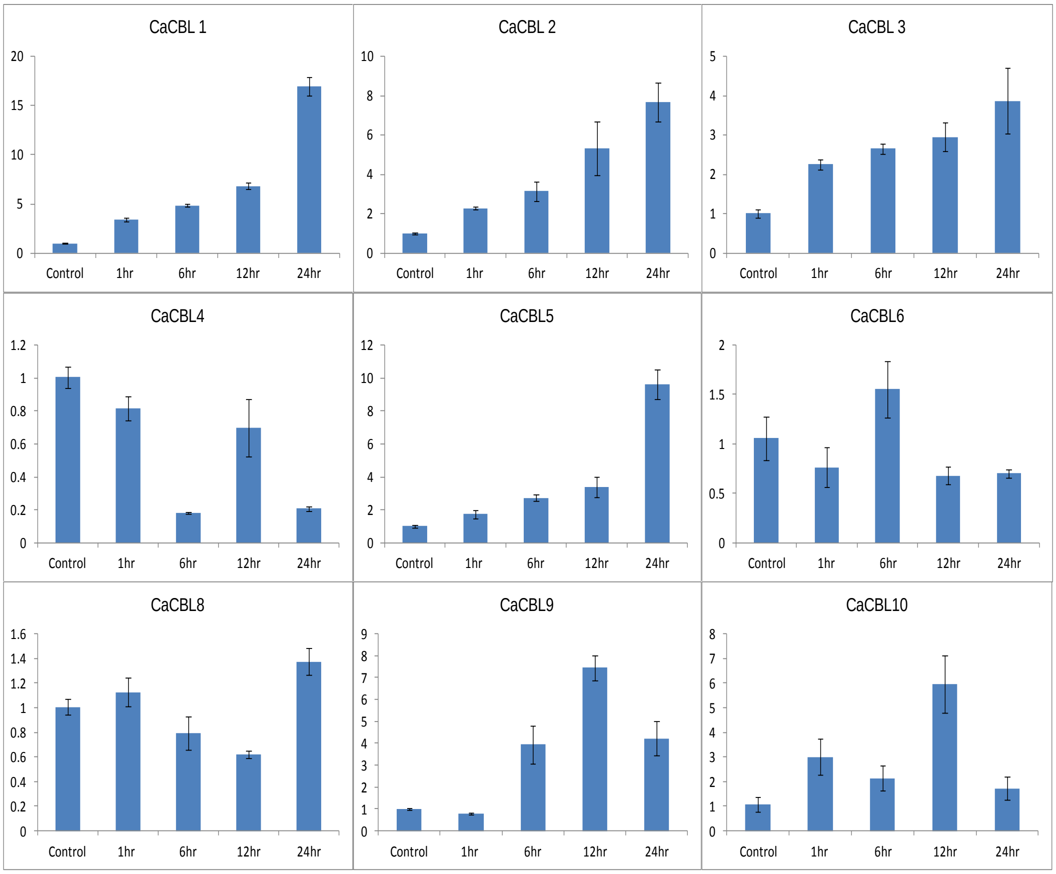

S4C Fig. Relative expression values of *CaCBL* genes in chickpea seedlings exposed to 250mM sodium chloride for different period analysed by qRT-PCR

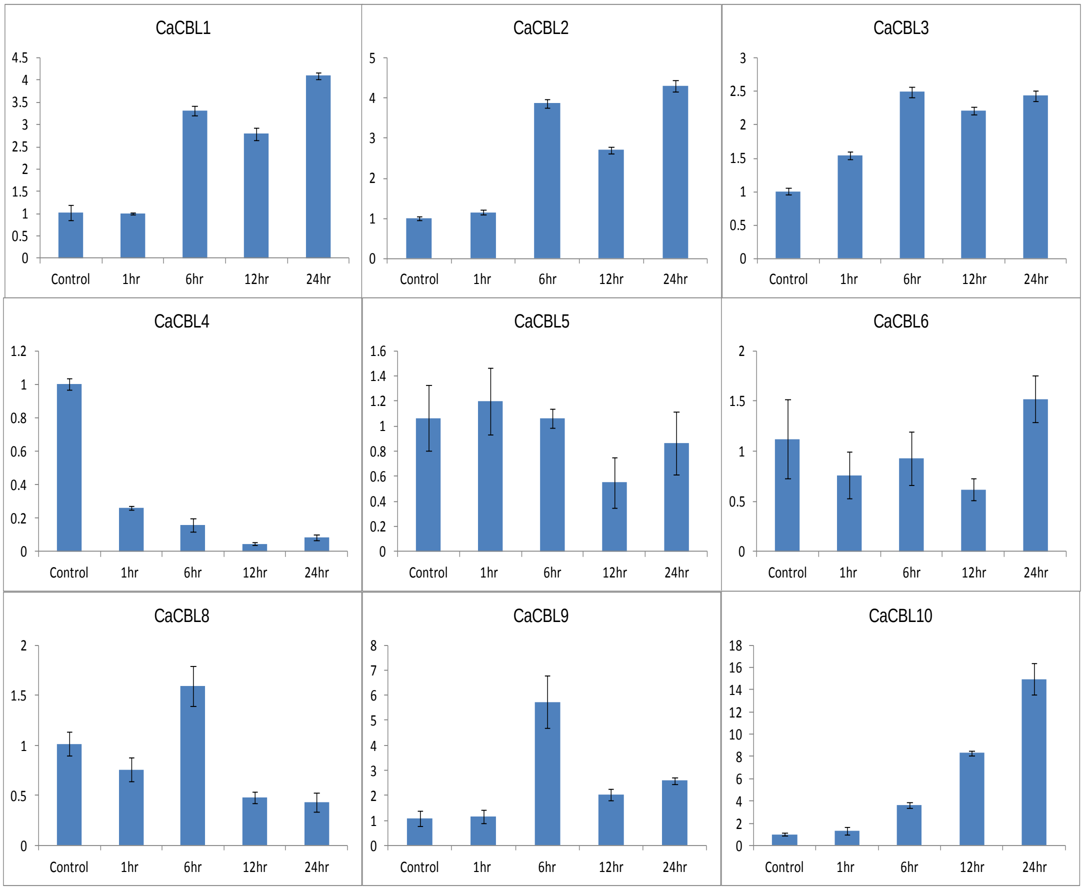

S4D Fig. Relative expression values of *CaCBL* genes in chickpea seedlings exposed to 4°C for different period analysed by qRT-PCR

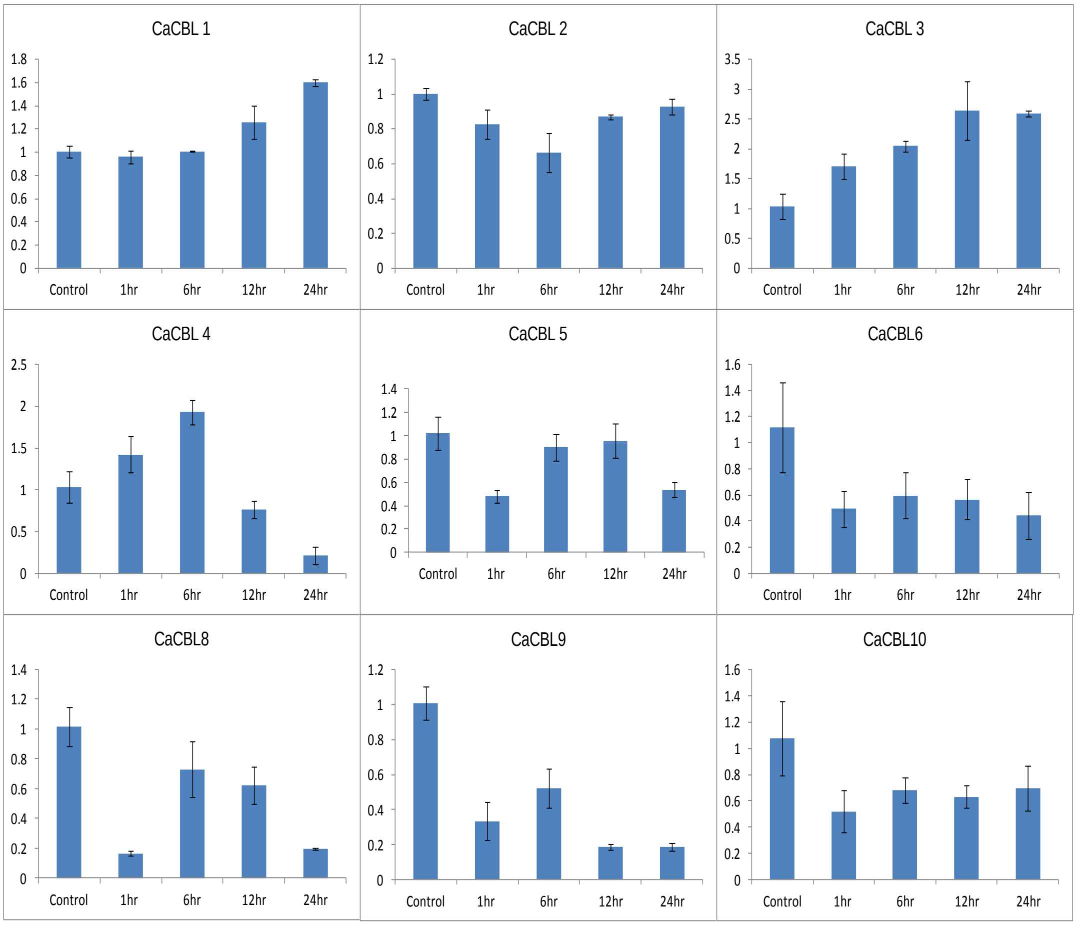

S4E Fig. Relative expression values of *CaCBL* genes in chickpea seedlings exposed to ABA (100µM) for different period analysed by qRT-PCR

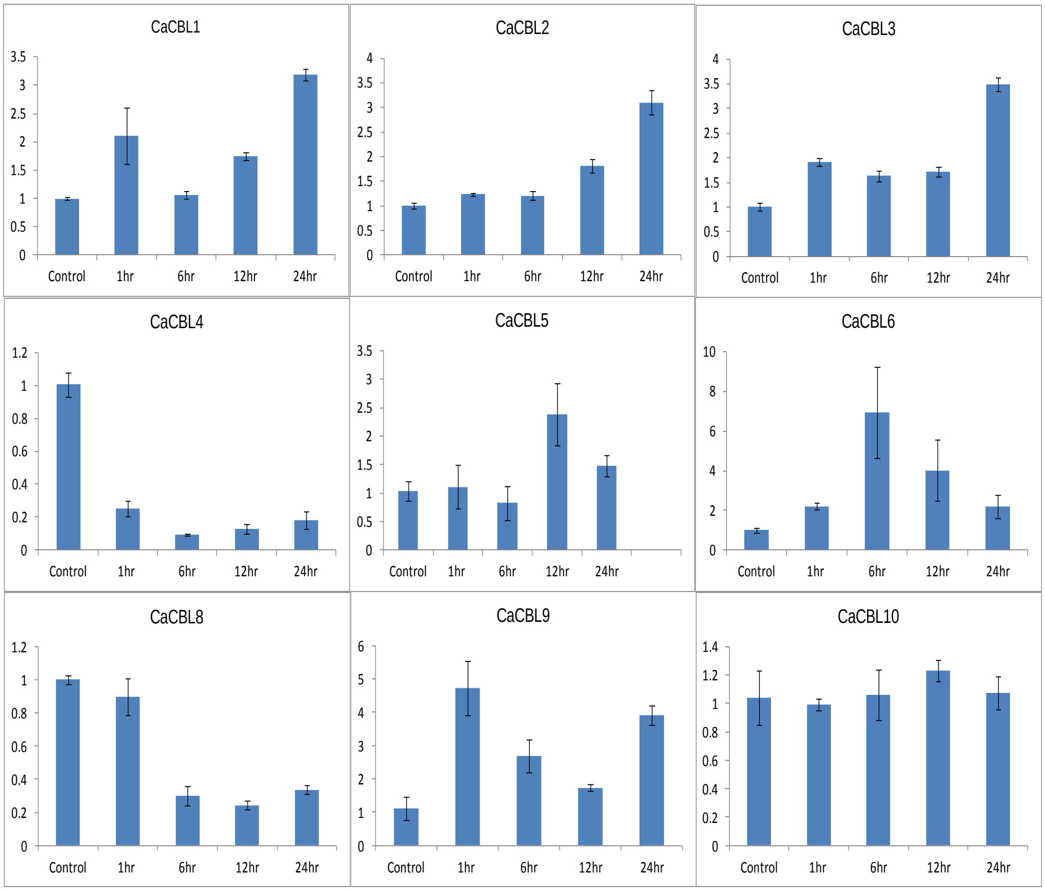

S4F Fig. Relative expression values of *CaCBL* genes in chickpea seedlings exposed to BAP (5µM) for different period analysed by qRT-PCR

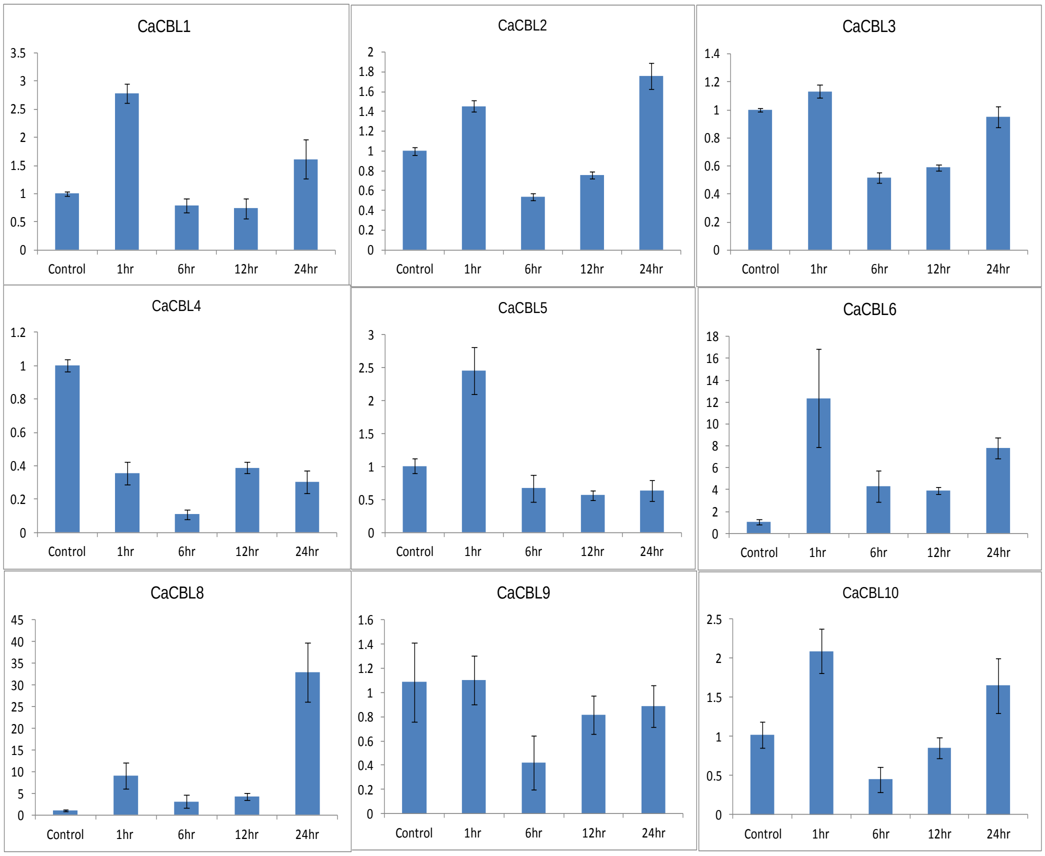

S4G Fig. Relative expression values of *CaCBL* genes in chickpea seedlings exposed to IAA (5µM) for different period analysed by qRT-PCR

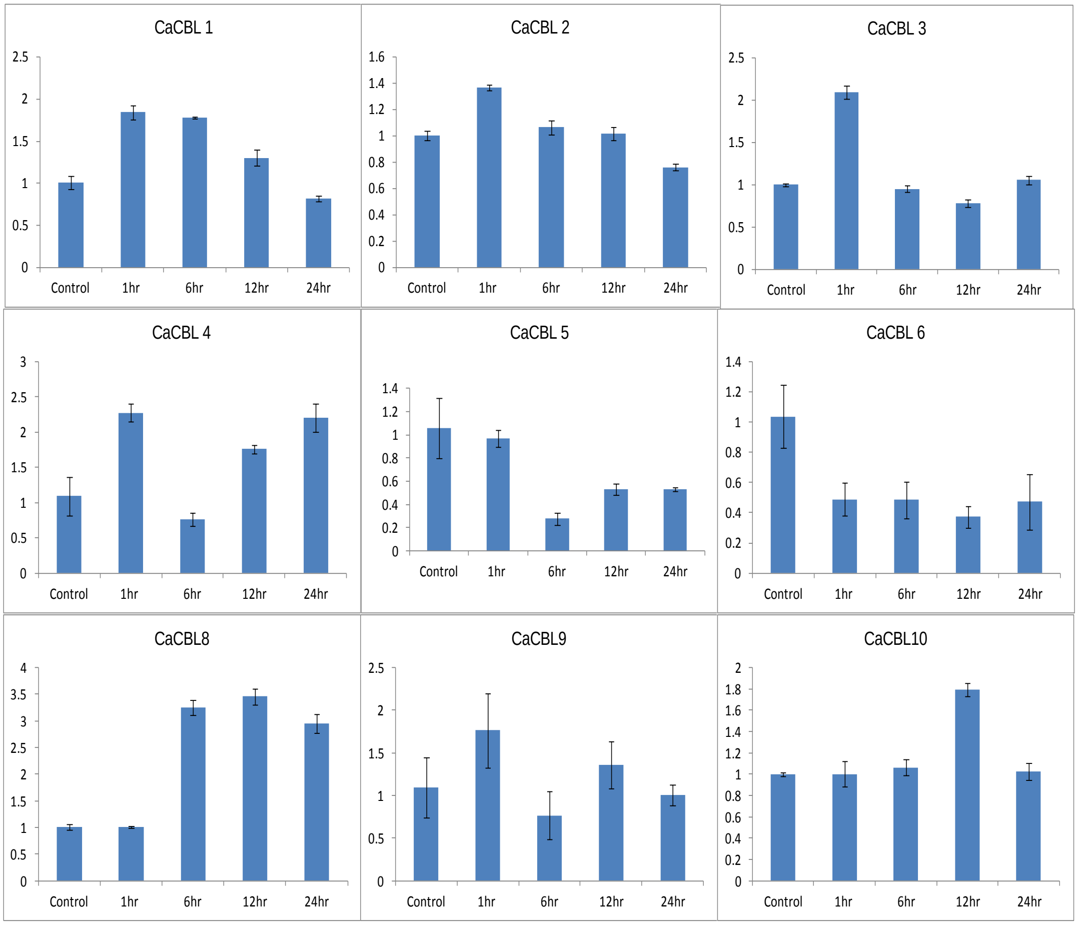

S4H Fig. Relative expression values of *CaCBL* genes in chickpea seedlings exposed to methyl jasmonate (100µM) for different period analysed by qRT-PCR

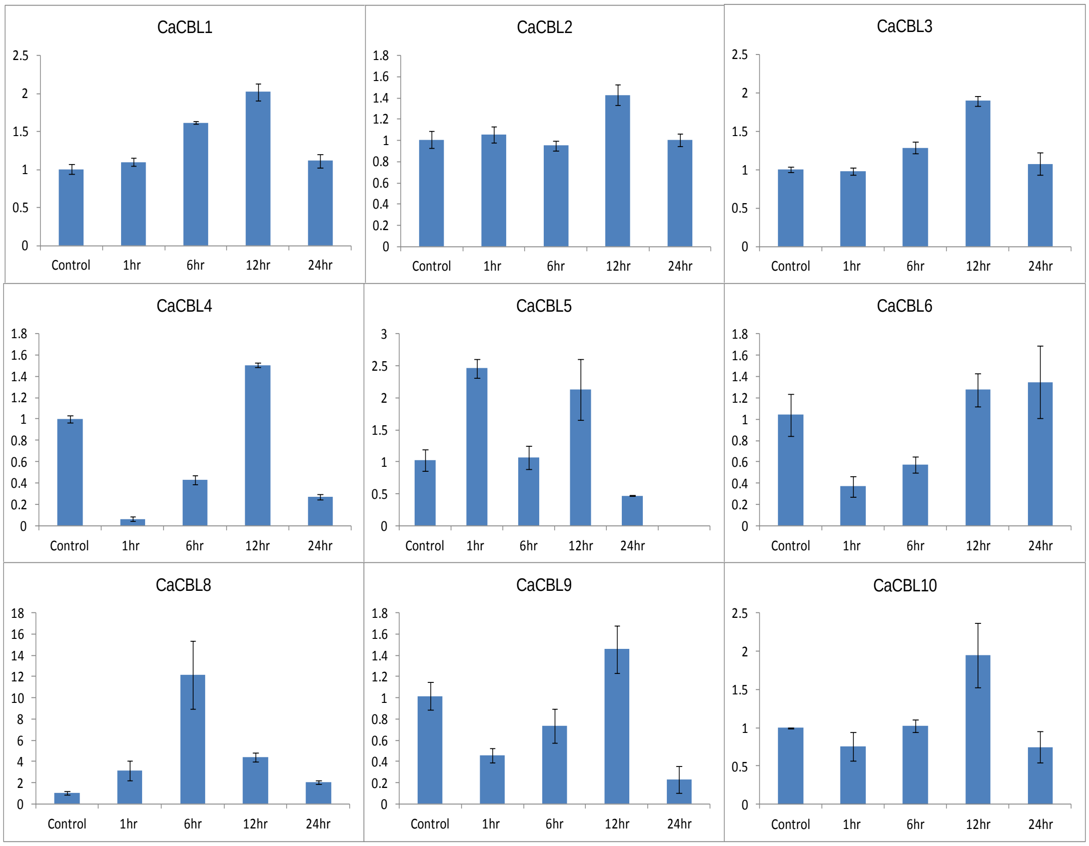

S4I Fig. Relative expression values of *CaCBL* genes in chickpea seedlings exposed to salicylic acid (100µM) for different period analysed by qRT-PCR

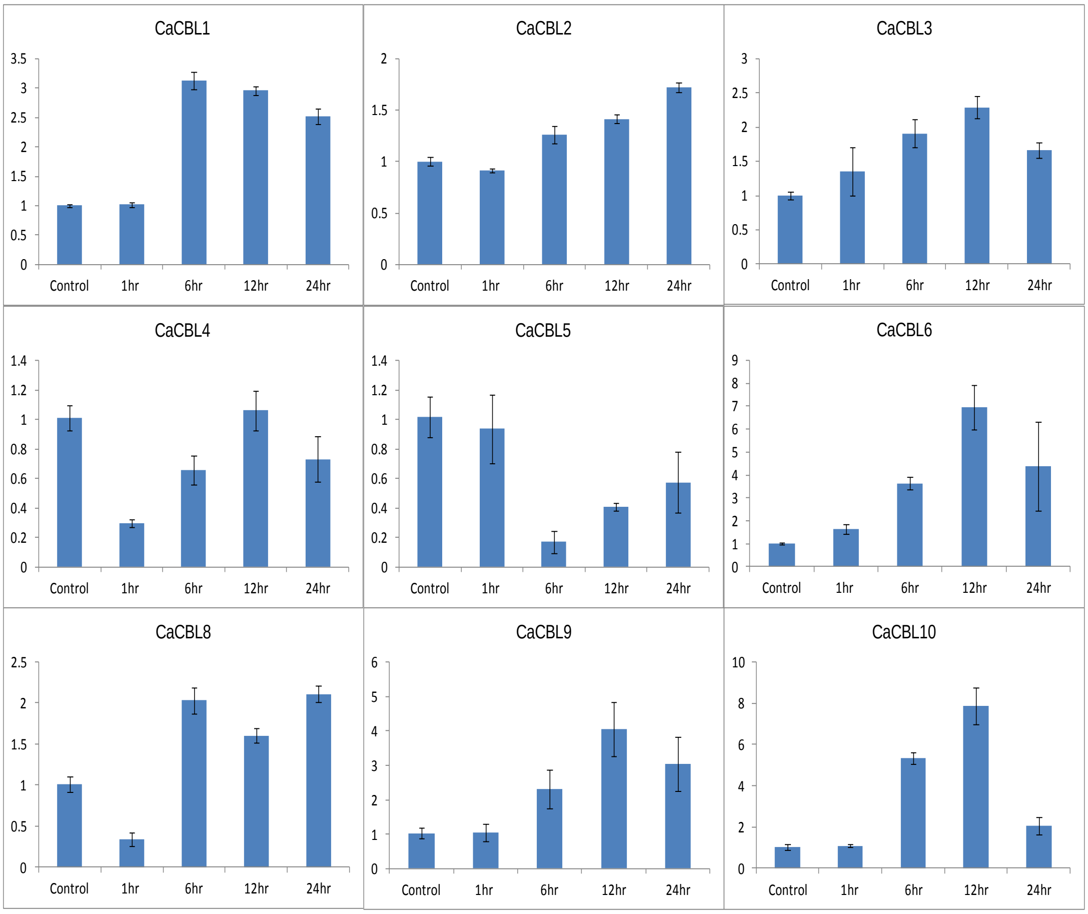

Supplement: S4 Fig — (PDF) [file pone.0123640.s004.pdf]
