# Supplementary material for: Investigation of Genes Encoding Calcineurin B-Like Protein Family in Legumes and Their Expression Analyses in Chickpea (Cicer arietinum L.)
Source: PLoS One. 2015 Apr 8;10(4):e0123640. doi: 10.1371/journal.pone.0123640 (PMC4390317; doi:10.1371/journal.pone.0123640)
Supplement: S1 Table — (DOCX) [file pone.0123640.s006.docx]

**S1 Table: CaCBL primers for cloning and sequence confirmation**

| CaCBL1F | CACCATGGGCTGTTTTAACTCTAAGCCA |
| --- | --- |
| CaCBL1R | AGCGACAATTTCATCCACGTTAGAATTAAA |
| CaCBL2F | CACCATGGTGCAGTGCCTAGACGGATTA |
| CaCBL2R | CTTCAGATACTGAAGAGTCATATTTTTCA |
| CaCBL3F | CACCATGTTGCAGTGCTTAGAGGGATTTAA |
| CaCBL3R | AGTATCATCTACTTGTGAATGGAATACAA |
| CaCBL4F | CACCATGGGTTGCTATTTTTCAACTTCAA |
| CaCBL4R | CAATTCTTGCTCTTCAACTTCTGTTCTTG |
| CaCBL5F | CACCATGGGGTGTTCTTGTACCAAAC |
| CaCBL5R | TGGAGTACTGATTGTGTCATCTTCAATGTC |
| CaCBL6F | CACCATGGTGCAGTTCTTAGACGTATTGAA |
| CaCBL6R | ATTTAACAAACTAGCAAGACTTTCATCCCT |
| CaCBL8F | CACCATGTCCATTTCAATGAGCTGCTTTTG |
| CaCBL8R | GTCTTCCACCTCAGTATGCAAAACAAAGCTG |
| CaCBL9F | CACCATGGACCACACTGTATCTCTGAGAT |
| CaCBL9R | GCCATTGACTTGCCAGTGGGAATCATC |
| CaCBL10F | CACCATGCCCACGGATTCCCCTGGTG |
| CaCBL10R | AAGTTCAGCCTCTGATTTGAAAATAAAACTAG |
