# Supplementary material for: Investigation of Genes Encoding Calcineurin B-Like Protein Family in Legumes and Their Expression Analyses in Chickpea (Cicer arietinum L.)
Source: PLoS One. 2015 Apr 8;10(4):e0123640. doi: 10.1371/journal.pone.0123640 (PMC4390317; doi:10.1371/journal.pone.0123640)
Supplement: S2 Table — (DOCX) [file pone.0123640.s007.docx]

**S2 Table: CaCBL primers for qRT-PCR**

| CaCBL1 RT F | AGACAGTTTCCTGGGAAGGAG |
| --- | --- |
| CaCBL1 RT R | CTGACAGTGAAAGCCGTCTG |
| CaCBL2 RT F | GTGCAGTGCCTAGACGGATT |
| CaCBL2 RT R | CAACAATTTACCACAGCAGCA |
| CaCBL3 RT F | TTGCGTTGTTGTGACATTGA |
| CaCBL3 RT R | CCCTTGCAAGAAGTTCAGGA |
| CaCBL4 RT F | CAAAGAAATCCAAAATTCCAGGT |
| CaCBL4 RT R | TCACAAGGTGTCTCAGAAGCA |
| CaCBL5 RT F | TGCAGTTTCGTGGTTTTGAA |
| CaCBL5 RT R | TCATGGAGTACTGATTGTGTCATC |
| CaCBL6 RT F | CAACTTTGTGCTGCTGTGG |
| CaCBL6 RT R | GTAAACCACCGGGCTGCT |
| CaCBL8 RT F | TGAGCTGCTTTTGCTTGATG |
| CaCBL8 RT R | TGTCTCCAAAGCAAGAAAGGA |
| CaCBL9 RT F | CACTGGCAAGTCAATGGCTA |
| CaCBL9 RT R | TCTGGCATGACCACATCATT |
| CaCBL10 RT F | TCGAGGTTCTGGTTTTTGCT |
| CaCBL10 RT R | GCGAAAAATGGAGAGTGGAG |
| EF1a RealT_F | TCCACCACTTGGTCGTTTTG |
| EF1a RealT_R | CTTAATGACACCGACAGCAACAG |
