# Supplementary material for: Investigation of Genes Encoding Calcineurin B-Like Protein Family in Legumes and Their Expression Analyses in Chickpea (Cicer arietinum L.)
Source: PLoS One. 2015 Apr 8;10(4):e0123640. doi: 10.1371/journal.pone.0123640 (PMC4390317; doi:10.1371/journal.pone.0123640)
Supplement: S3 Table — (DOCX) [file pone.0123640.s008.docx]

**S3 Table: Physical properties of CaCBL proteins**

| Protein | Peptide length (aa) | Molecular weight | PI | Negatively charged residues | Positively charged residues | Instability Index |
| --- | --- | --- | --- | --- | --- | --- |
| CaCBL1 | 213 | 24469.9 | 4.66 | 36 | 24 | 34.71 |
| CaCBL2 | 208 | 23863.2 | 4.85 | 38 | 25 | 39.49 |
| CaCBL3 | 226 | 25987.5 | 4.7 | 41 | 25 | 39.65 |
| CaCBL4 | 222 | 25593.1 | 4.67 | 41 | 26 | 42.33 |
| CaCBL5 | 214 | 24378.7 | 4.82 | 38 | 25 | 38.56 |
| CaCBL6 | 226 | 25694.2 | 4.68 | 36 | 20 | 37.77 |
| CaCBL8 | 215 | 24682.5 | 5.03 | 37 | 27 | 40.71 |
| CaCBL9 | 258 | 29603.6 | 4.67 | 46 | 27 | 41.72 |
| CaCBL10 | 252 | 28413.4 | 4.57 | 42 | 23 | 38.76 |
