# Supplementary material for: Investigation of Genes Encoding Calcineurin B-Like Protein Family in Legumes and Their Expression Analyses in Chickpea (Cicer arietinum L.)
Source: PLoS One. 2015 Apr 8;10(4):e0123640. doi: 10.1371/journal.pone.0123640 (PMC4390317; doi:10.1371/journal.pone.0123640)
Supplement: S4 Table — B. Identification of cis-acting elements in 1.2 kb promoter regions of CaCBL genes. (DOCX) [file pone.0123640.s009.docx]

**S4A Table: Qualitative expression profiling of *CaCBL* genes in different tissues and in response to different treatment based on qRT-PCR result (**↑,↓ and – denote upregulation, downregulation and no change in expression).

| Name | 20% PEG | Salt | Cold | ABA | BAP | IAA | SA | MeJ | Tissue specificity | Vital role |
| --- | --- | --- | --- | --- | --- | --- | --- | --- | --- | --- |
| *CaCBL1* | ↑↑ | ↑ | - | ↑ | ↑ | ↑ | ↑ | - | F>L>S>R | Abiotic stress, development |
| *CaCBL2* | ↑ | ↑ | - | ↑ | - | - | - | - | F>L>S>R | Abiotic stress |
| *CaCBL3* | ↑ | ↑ | ↑ | ↑ | - | - | ↑ | ↑ | F>L>S>R | Biotic, Abiotic |
| *CaCBL4* | ↓ | ↓ | - | ↓↓ | ↓↓ | - | - | ↑ | F=R>L>S | - |
| *CaCBL5* | ↑↑ | - | - | ↑ | ↑ | ↑ | ↓ | ↓ | F>S>L>R | Abiotic stress, development |
| *CaCBL6* | - | - | ↓ | ↑↑ | ↑↑ | - | ↑ | ↓ | F>L>S=R | Defence, development |
| *CaCBL8* | - | ↓ | ↓ | ↓ | ↑↑ | ↑↑ | ↑ | ↑ | R>L>F>S | Defence, development |
| *CaCBL9* | ↑ | ↑↑ | ↓ | ↑ | - | - | ↑ | - | F>L>S=R | Abiotic stress |
| *CaCBL10* | ↑ | ↑↑ | - | - | ↑ | ↑ | ↑ | - | F>L>R>S | Abiotic stress |

**S4B Table:** **Identification of *cis*-acting elements in 1.2 kb promoter regions of *CaCBL* genes**

| *CaCBL1* | ACGTATERD1, ARR1AT, CBFHV(DRE), CPBCSPOR, DRECRTCOREAT, ERELEE4, GAREAT, GT1CONSENSUS, MYCCONSENSUSAT, POLLEN1LELAT52, SURECOREATSULTR11 |
| --- | --- |
| *CaCBL2* | ABRELATERD1, MYCCONSENSUSAT, POLLEN1LELAT52, MYB1AT |
| *CaCBL3* | GT1CONSENSUS, MYB1AT, MYCCONSENSUSAT, POLLEN1LELAT52, WRKY71OS |
| *CaCBL4* | GT1CONSENSUS, POLLEN1LELAT52, WRKY71OS |
| *CaCBL5* | ARR1AT, ERELEE4, MYB1AT, MYCCONSENSUSAT, POLLEN1LELAT52 |
| *CaCBL6* | ARR1AT, CPBCSPOR, ERELEE4, GAREAT, GT1CONSENSUS, POLLEN1LELAT52, WRKY71OS |
| *CaCBL8* | ARR1AT, ERELEE4, GT1CONSENSUS, WRKY71OS |
| *CaCBL9* | ABRELATERD1, ASF1MOTIFCAMV, CBFHV(DRE), ERELEE4, GAREAT, GT1CONSENSUS, MYB1AT, MYCCONSENSUSAT, POLLEN1LELAT52, WRKY71OS |
| *CaCBL10* | ABRERATCAL, ERELEE4, GAREAT, MYBGAHV, MYCCONSENSUSAT, GT1CONSENSUS, POLLEN1LELAT52, SURECOREATSULTR11, WRKY71OS |

| ACGTATERD1 | Dehydration responsive elements |
| --- | --- |
| ARR1AT | Cytokinin response regulators |
| CBFHV(DRE) | Dehydration responsive elements |
| CPBCSPOR | Cytokinin responsive elements |
| DRECRTCOREAT | Dehydration responsive elements |
| ERELEE4 | Ethylene responsive elements |
| GAREAT | GA responsive elements |
| MYCCONSENSUSAT | dehydration-responsive |
| POLLEN1LELAT52 | pollen specific activation |
| SURECOREATSULTR11 | SURE contains auxin response factor (ARF) binding sequence |
| WRKY71OS | Pathogenesis-Related elements |
| ABRELATERD1 | ABA responsive elements |
| MYB1AT | Dehydration-responsive elements |
| GT1CONSENSUS | Salicylic acid responsive elements, Light responsive elements |
| ASF1MOTIFCAMV | Salicylic acid responsive elements |
