# Supplementary material for: Investigation of Genes Encoding Calcineurin B-Like Protein Family in Legumes and Their Expression Analyses in Chickpea (Cicer arietinum L.)
Source: PLoS One. 2015 Apr 8;10(4):e0123640. doi: 10.1371/journal.pone.0123640 (PMC4390317; doi:10.1371/journal.pone.0123640)
Supplement: S1 Text — (DOCX) [file pone.0123640.s010.docx]

**S1 Text: Corrected sequences of *CaCBL* genes, CDS and proteins**

**CaCBL1**

Gene sequence

ATGGGCTGTTTTAACTCTAAGCCAACAAGACAGTTTCCTGGGAAGGAGGATCCAGTGATTCTTGCATCACAGACGGCTTGTAAGTCAGATTTTGTTTTTTAGTAAAATGATGTTATACTTCAATTTTTAGTAGTTATAAGATTCTGGGTTGTTGTGATTGGATCTGTTCACCAAGTTTGGAGTGTACATGTTTATGTGGAGCTGCTTATCCTGAACTTACATGTGGTTTTGCACTCTTTCTAAATGGATCTTAAAAGTATTATAATAATGATAAAAATAAGTCATTGCTGGTTGGCAAGGTAGTTCACTTTGTTTGGTTTATTGATAGAAGTAGGCAAAGATGGACATGTAATAAGGGCCTACCATCGTTCTGGCACAATTTAAACCTAAATAGGCTGCTTTCTCTCCCATTTTCTACCAGATTTTTATCGCTCTGGCACAATTTTAAACCTATGTATTCAGCCAGCTCTACCAGTTTAAGAAACAAAATCGTGACTTGTTGGTTCATATGGTCAACACCTCAAATGAAATTGATAGGATTTGAGACACTTGTGTTTGTAGTTGTTTAAACTTACTTGTTATGGAATTACTCTCTCTATTTCCCAAGCACTCAAGTTAGTAGTTTAAACTTATGTATCATATTTTTGTTTTCAGTCACTGTCAGTGAGGTCGAAGCATTATTCGAGCTGTTTAAGAGCATCAGCAGTTCTGTTGTTGATGATGGACTAATAAGCAAGGTACTAGATAGCAACTCTGCATAAATATTTTTAGGGAGTTTATGAAAATATGCGGAAAACAATTTATGGATATATCTTAAGCTATTGCCACAAGCTCTTACAAACACTCTCATAAGATAATCCCAAATAAGCTCTTCTAAATGCCTCCTAATCAAGATAACATTTATGCTTATTTGTTCTTCACATGTAACTTGTATCCCGCAATGTGATTATGGGTATTCTTTCTTTGCAGGAAGAATTTCAATTGGCAATCTTCAAGAATAAGAAAAAAGAAAACATCTTTGCAAATCGGGTACTATATATCATACTTTTACTTTCTTAATAACTAACTATGCCGTAATTGTTTGTTCCCTGCACGCATGTCTAATGCATGTAAGCAGCCCTAAATGTACTTTTAGATCAGCAAATTTCTTCAAGCAGTTTATTGAAGAACTTTGCTGTTTCTTTCACTGAGTTTTTCTTGAAGAAACTCAATAGTACTTTTAAGACGAGCATGAAGCATTTTCGTTGTGAACCATTTGAATGAATTCGGTATTTGTCAACTTTATATACGTAGAATTTTGGAAACCACGGGATCATAGAGTTAAAGATGTATCTCATATTAAAAATATGCTCGAGAGCATGATACATTAGTATATTCCTTAGAATATTTCATATAGTAAATGGTACAATGAAACACTTATTTATAGCCTTGTGTTCCCTAACTAAGTTCTTAACCTCCAAACTAACTTCTTAACAAACTTATGCTAACTTACTCTAGAATTATATTCTCTAACATAGGATGCTTTTGTCAATTCCTTATTTTTGTGTAAGATAGAAATCAGTGGTTGATTAGCCTTGCAACTTTCTTACTTACTTCTTGGCTAACATATTATGTAATTCTTATGAAATAACTTTTTCAATTGACTGGATATCTATTGATACGATGTCAGAAATGTATACATGCAGATCTTTGATCTATTTGATGTCAAAAGGAAGGGGGTTATTGATTTTGATGACTTTGTTAGATCTCTCAATGTTTTCCACCCAAACGCACCACTAGAAGACAAGATAGATTGTGAGTTACAAATTGAATCCTCGACTCATTGTGGTTTACCTTAAATTCCTTACTTTGTTAGATTTAGCTGTCCTGATATATTAATTAAGTTTGGGATTTTCCTGCAGTCTCATTTAGGCTCTATGATTTGCACAACACTGGCTTTATAGAGCGTCAAGAGGTAAGATGGTGATTATCTGTTCTATGTCTTCAATGAAAATGAAATGCTTGCTAATCCACCGAGAGTGGTGTAATGAAAATTTTCGTTTAGGTCTTGTTCTTCAGACCATTCTTTGTAAGAAGATAGATTGCTATGACATGTATGCTCTTTCTCTGTGCACTGATATCCTTGGCTTTTCTTTCCCTTTGTTGGTGATGTCTGACAGGTCAAGCAAATGTTAATTGCACTTCTTTTCGAGTCTGAAATGAAGTTGGCTGATGATGTGATAGAAACAATTATAGACAAGGTCTGTTTGCAAAATCTGTTGATAACATAAAATATTATATGTGAAAATCCATCTGCAGAAGAAATATTTTTTTTTGTTATAGAAGCTGTGCTGTAACCTCTTTTTCATCAAGATGATTGCAAATTTAATGAAAGACTTGAGCTTGAAGTTTATTTTTCTGGCCAGACTTTCTTGGATGCTGACACGAACCAAGATGGAAAAATAGACATTGTCGAATGGCAGAAGTTTGTTTCTGAAAATCCATCACTGCTCAAAATCATGACCCTACCCTACCTAAGGTATGTGCTGGATTGTTTTCATGGTAAATATTAAACTACTTGTATCCCTATTTTGATGTTCAAACTATAGTATAATTTGTTATTCTTGTCATTGATGCAGGGACATAACGACTTCTTTCCCAAGTTTTGTATTTAATTCTAACGTGGATGAAATTGTCGCTTGA

CDS

ATGGGCTGTTTTAACTCTAAGCCAACAAGACAGTTTCCTGGGAAGGAGGATCCAGTGATTCTTGCATCACAGACGGCTTTCACTGTCAGTGAGGTCGAAGCATTATTCGAGCTGTTTAAGAGCATCAGCAGTTCTGTTGTTGATGATGGACTAATAAGCAAGGAAGAATTTCAATTGGCAATCTTCAAGAATAAGAAAAAAGAAAACATCTTTGCAAATCGGATCTTTGATCTATTTGATGTCAAAAGGAAGGGGGTTATTGATTTTGATGACTTTGTTAGATCTCTCAATGTTTTCCACCCAAACGCACCACTAGAAGACAAGATAGATTTCTCATTTAGGCTCTATGATTTGCACAACACTGGCTTTATAGAGCGTCAAGAGGTCAAGCAAATGTTAATTGCACTTCTTTTCGAGTCTGAAATGAAGTTGGCTGATGATGTGATAGAAACAATTATAGACAAGACTTTCTTGGATGCTGACACGAACCAAGATGGAAAAATAGACATTGTCGAATGGCAGAAGTTTGTTTCTGAAAATCCATCACTGCTCAAAATCATGACCCTACCCTACCTAAGGGACATAACGACTTCTTTCCCAAGTTTTGTATTTAATTCTAACGTGGATGAAATTGTCGCTTGA

Protein

MGCFNSKPTRQFPGKEDPVILASQTAFTVSEVEALFELFKSISSSVVDDGLISKEEFQLAIFKNKKKENIFANRIFDLFDVKRKGVIDFDDFVRSLNVFHPNAPLEDKIDFSFRLYDLHNTGFIERQEVKQMLIALLFESEMKLADDVIETIIDKTFLDADTNQDGKIDIVEWQKFVSENPSLLKIMTLPYLRDITTSFPSFVFNSNVDEIVA

**CaCBL2**

Gene sequence

ATGGTGCAGTGCCTAGACGGATTAAAGCATTTGTGTGCTGCTGTGGTAAATTGTTGTGAATCTGATTCAATAAAGCAACCTAGAGGTTTAGAGAATCCAGAACTTCTTGCCAGAGAGACAGTGTGTATGTTGTTATTTTCCCTATTTTTTCATTCCATATTATGTATGCTGTAAGGAACTTTCCAATGAAGCAATAGATCAGTCATTCATTGTTGTCTCAAATATGTCAATAGTAATTTGATTTAATATTTTCTTGCATCTAATTGTATCCATATCTCCCTATTTGCTTTTTTTCACAATATTTTGGAAGATGGCAGTTGAAAAATATCAAATACTTGAAATGAGCATCATTATACTGTAGCATTAAACTCTATAAATAGTGTTGTGACTTACTTGAATAACCCTATTGTTCAGTTATTGAAGCATTTGTTGAGACTTGATTCACTTGAATAAATATTGATATCTCTCTCAAGTTTCTTCTTTTTATGAAAAGCTTAAGGAAATGGAAAGAATAAAATAAAGCACAAAGAGAGGATATTTATCCGTTTTATTGCCAATGCAGTTAGTGTAAGTGAGATTGAAGCACTTTATGAACTGTTCAAGAAGATCAGCAGTGCAGTGATTGATGATGGACTAATTAATAAGGTGAGCTATGATATATCATGCTCTATTATTCTATCAATTCGTGTTATGATATGTGTATTTTCCACCCTTATAGATGATGGTTTTCATCCTTGCCTACCCTATAATCGTACAAATCTACATGCTTTATGTCTAACACATAACTCACAGTAACTTATATGATCTATTGTATTTTTGTTGTTCATAGTTCTAAACTTTTCTTTTTGTTTTTACGACGGTTTCTTTATTTTACAGGAAGAATTTCAGTTGGCGTTGTTCAAGACAAACAAGAAGGAGAGCTTGTTTGCAGATAGGGTATGCTGAGCATTATCTACTTCTCTTTACAACTCCTTTTTTTTAATTTCAATCTTATGGAAAAGAAACAATAATATGATCTTTTTTGGGCCTTCATTGTTTTTCCCCACTGATTTATTAACTAGATGGTCCTTTTTGGGGCAATATTTATGATCTGATATGACTATGAAATCAGCTGCACACTGCATAGATAGTGTACTATATGATGTGAACCTTATAAATTTATTTATCCTGTTCGTGATTTTATACTGTTTGAATTATATAGGTGTTCGATTTATTTGACACAAAGCACAATGGGATACTTGATTTCGAAGAGTTTGCACGCGCTCTCTCTGTCTTTCATCCAAACGCACCAATTGATGATAAGATTGAGTGTAAGAATGACAACTATTTAATCAACAATTTTATTTGGATATTGAATGGAAAGCATATTCATTGGAGAGAGAGAGGGGAGGGGGGTTACTAGGTAGAACATATATGTTGTTTCTACACTTGTCTATTTATTTCCCTTACCGTGTTATTATTGGTAGTAATGCATAAACTTGTTGGGAGATTCCCAACTTTTCCTTCTTGCAGTTTCTTTCCAATTGTATGATCTCAAACAGCAAGGTTTTATTGAAAGGCAGGAGGTAAATTTTTAATGTTCGTATCAACTTGAATACATGTAGAAGTTTTGTTCACTCGCCACGTTGAACCAGCCTTGACTTGCATAATTTATTTTTCTGAAGCCCACGGAAGGATTTACCCTAGGTCAATAGGAGTAAGCAAGTTTTGAATCATAGATGATAACCTGTTCAGGAATTAAACAATAGTGTTATTTTATATAAGGTTGCTAGTTTATGCACACTCATTTTCTAGTGGGTGTAAATTAGCATCCTACACCTTAACTTTTTATTATAAATTGCTAATTTACACCACCTAGAAAATGAGTGGTGCAAGTTATCAAATTGCTAACTTGCACCACTCATTTTCTAGTTTATGTAAATTAGCAAGCTACACTTTAAGGTGTATTTCACAAATTTGTGGCTATAACTTGCTAATTTACACCCAATAAAAATTAAGTGGGTGTAAATTAGCGAACCCCTTTCGTTTATAATAATATTTAGGTACCTTATTTCAAGACAGAATTTGACAACCTAGATTTGATATGGAGTGATGATCAATGTTTTTAAGTGTAGGTAAAACAAATGGTGGTGGCCACTCTAGCTGAATCTGGTATGAACCTTTCAGATGATGTGATCGAAAGTATTATTGACAAGGTGCTTCATCTACTTGACAACTTAAAGTGCATGCCAAATCTTTATGATCATTATTAGACTAGGGTACATTTGAAGCTTTTTTTTTTCTGCTTTTTTATTTTTTTATGTTATTTTAGTTTTTTTGTTGGTTTAAGTAAGAAATTCATGAATTATTTTTTCACTCTTATTAAAAGTCTCCAAAAGCAATCTTATGCAAGCATGTTAACAAATTGTATGCAACACATGCTGCGTGCATAGTTTGGAATTCAGCAGTTTTATGTTTCCTTGTTATAATTGATTTTCCATTGTTTCCAAATTGTGCGCTTTTGAAGGTGCATCTTTTTCTGTCATCGTTTCTGACAGTTAATTAGTGAATTATTAGCGGGATACTTATGTGTACAATATGTTTTGTTTCCCTCGCATTCAGACCTAAGCTTCCACTAAATCTGTCTGTTAATTAAATTAAACCTTCTTTGAGCGTTGTTCTTGTGAACTTCATTTGTTGACTAAGATTTATGTGTGTCGAGTTGATAATGAGGACATTGGCATATGATTTCATTTTTGTGCTATTATAATCTTTTCTGGAGATTTGTCACTAACATTTTCTAAGCTATTTAGACATTTGAGGAAGCTGATACAAAACATGATGGGAAGATTGACAAGGAAGAATGGCGAAACCTTGTCTTGCGGCATCCATCCCTTCTGAAAAATATGACTCTTCAGTATCTGAAGTGA

CDS

ATGGTGCAGTGCCTAGACGGATTAAAGCATTTGTGTGCTGCTGTGGTAAATTGTTGTGAATCTGATTCAATAAAGCAACCTAGAGGTTTAGAGAATCCAGAACTTCTTGCCAGAGAGACAGTGTTTAGTGTAAGTGAGATTGAAGCACTTTATGAACTGTTCAAGAAGATCAGCAGTGCAGTGATTGATGATGGACTAATTAATAAGGAAGAATTTCAGTTGGCGTTGTTCAAGACAAACAAGAAGGAGAGCTTGTTTGCAGATAGGGTGTTCGATTTATTTGACACAAAGCACAATGGGATACTTGATTTCGAAGAGTTTGCACGCGCTCTCTCTGTCTTTCATCCAAACGCACCAATTGATGATAAGATTGAGTTTTCTTTCCAATTGTATGATCTCAAACAGCAAGGTTTTATTGAAAGGCAGGAGGTAAAACAAATGGTGGTGGCCACTCTAGCTGAATCTGGTATGAACCTTTCAGATGATGTGATCGAAAGTATTATTGACAAGACATTTGAGGAAGCTGATACAAAACATGATGGGAAGATTGACAAGGAAGAATGGCGAAACCTTGTCTTGCGGCATCCATCCCTTCTGAAAAATATGACTCTTCAGTATCTGAAGTGA

Protein

MVQCLDGLKHLCAAVVNCCESDSIKQPRGLENPELLARETVFSVSEIEALYELFKKISSAVIDDGLINKEEFQLALFKTNKKESLFADRVFDLFDTKHNGILDFEEFARALSVFHPNAPIDDKIEFSFQLYDLKQQGFIERQEVKQMVVATLAESGMNLSDDVIESIIDKTFEEADTKHDGKIDKEEWRNLVLRHPSLLKNMTLQYLK

**CaCBL3**

Gene sequence

ATGTTGCAGTGCTTAGAGGGATTTAAGTCTTTACTCGCTTCTGTATTGCGTTGTTGTGACATTGATATATATAACCAGGCAAGAGGCCTTGAAGATCCTGAACTTCTTGCAAGGGAGACCGTGTGTATGCCATTTAACGTTGCTTTATTCAATTGATTTGTTACTTGCTACTTCTACTTTTACAAAATTAGATTGCTATGAAGCACTGACACAGACACCGAACACGACACCGATGCGATGACATTGATAATAATTGAAAAATAGAAGAAATCGAATGTAACTACACCTAACACGCCTTCAATATAAAGTGTTGGGGATACACAAGATTACGAAAGAATTTTAATGTCAACCTCTGTGAAGTGTCTGATGATTGAGAATATTTTCTTTCGAAATCCTGTATCTTGTGTTATTCCGTTGAGTTTTGAGGTAATGTAATTAGTGTGATGAACTAGAACAAAAAAGAAACATGTCATTATCTCTTTGTATTGCCGATTTTATTACCTTTGACACCGCTGTTTTGGAAATAAATTTGCAATATTTGTACTGAAGGTGGCTATTTATTTTGCATATTTATGCAGTCAGTGTTAGCGAAATAGAAGCACTGTATGAGCTCTTCAAAAAGATCAGCAGTGCTGTAATCGATGATGGGCTGATTAACAAGGTTTGTCTTCTTTGTCATTGGACAACTTATGTTTGGCATATTATATTTCATCGATGAAACATATTCTCTTAATGAAAGAGAAAAGTGTCACATTTGTTGTTTTATTTATCATTCTATATCAATTTGACAAATTTTATTTTTTATTTCATCGGTTTAGTTTAAAAAAAATATTAGATGTTATATTCAATTTTAGAGTTCATTTTGCAGGAAGAGTTTCAATTGGCATTATTCAAGACGAATAAAAAAGAAAGTTTGTTTGCTGATCGGGTATTGCTTTCCCTTCCATTTTATCTGTTTTTGTGCAAATATCTTTACTTTAAAGCTAAACAATTTATTTATTTATTTATAATATCAGGTTAATATCCATTTATTCATACATTTATATTAGAAGAAAGTGTTCCTGTATACTGTTAGATGACTCAAATTTTGGCTGTCCTCGATTTTATTTGTTTTTAATGATTGGTTTGGCTGCTCTGGTTTTGAATAGAGTCACTACTCTATTCAACAAATTCAGAATATTCAAAAGAAGATCTCTAACCAACTACATGGCTCCATATTTATACACAATAGACCTAACAACACATAACTAAAAGACTAACTATTAGTAACTAACTGTACTAATCTAATTCTTAACATTATGCCAATAGATAAGCTCAAATAAGTCAATCCAAACATACCTTTTATCAATCTCCTTGCCTAGTCATTTCCAAATATTCTATTGACCCCCTACTTCATTTTTTCTTTGTGTGTGAAATAGTTGGAGAATGTTAGGGCAATTTTGTCGGTTCTCATTGTGATTTATACTTGCTGAAGATATTTTTATTATGGGGCTTTCTTTTCTATTTGGATCATCAATTGAGATTGAAGCAAGTAGTTATTTTAGGGGTAATCCAGGAAGGGGTTAAATTTACATTTAAAACCGGTATATGGTGTGGATTATACTAGCTTGAATATTTTATTAAACAATAAAGATAAGAATCATTAAGATTTTGTTCAACTTTGTACCTCAAAGGTATATCGTTTTAAAAGTCTGGGAAGGTATGAGTGTGCGTGTACGTTTTTGTAGGCGTAACTAAGAAGTAAGATGACAATATATGATAAGGTTCCGAGTTAGTTAATTTACCGGAAAAATTAGTGTATGCAAGTTAGGGTGCTACAACCTCTGCCTTATTATACAATGTTTACTATCATGTTCATATGTTTTGTCCGGCCAATTTAAATATTATTTCGACATTCTGTTGTTCTTTTTTTGTAACATGTAGGATGACTTATTCATATTACTCCATGTGGTTAATATCTCACTCGAGTTTCTCTCTAACTCTATTAGGTATTTGATTTATTCGACACAAAGCACAATGGGATCCTCGGTTTTGAAGAGTTTGCTCGTGCCCTATCCGTTTTCCATCCAAATGCCCCTATTGATGATAAGATTGAATGTATGTTGTACTTCAGCTTCTAAAATTCTAACATGAATGCACATTTGGAGTCAGTTCTTTAATTTACATATGTAAAAAATTCTTTAACTTACAAATGTCATCCTTGTCTTTAAATAAATGAGAACTAAACCAATATAATATCACATTTCAGTTTCCTTTCAACTATATGATCTCAAGCAGCAGGGTTTTATCGAACGACAAGAGGTACGTCATCTTCTTATATCCAATTCCTACTTAAGTATTCTGATCCTCATTTGAGACAAGCTTGATTTTTGTCTGTGTTATTATATAAAGGTGAAGCAAATGGTGGTTGCTACCCTTGCAGAATCGGGTATGAATCTATCGGATGATGTTATAGAAAGTATAATCGACAAGGTGCTTCCTCATTCTTTTGTCATGCACTTCTAATCTCATACAAGAATTTGAGTCTCTTTTTTTGAAAATCCACTTGTATAGTTTTTTCAACATTAAATTTGTGTATTATTTCAGACTTTTGAGGAAGCAGATACAAAGCATGATGGCAAGATTGACAAGGAGGAGTGGAGAAACCTTGTTTTGCGCCATCCGTCACTTTTGAAGAACATGACCCTTCAATATCTCAAGTAATTTTTCAATCCATATTTTTCTGATAATTTTTAGTGGATGGAGTGGAGTGTCAAGCAACAATCTTTCTAACACTCTCTGCTATTGTTTAAAATTCAAACAGAGTTCCCCCTAAATTATTTGGGTACCACTTGAATTTAGTATGAATTTGAACTAATAGAAGAGTATGTTTCAGAGTGTGTTGCTAGCATAATTCTTTTGTGGAAAATTTCTCCAAAATGGAGACAACCAAATAACACTGTCTTTAGAATTTTTTGTTATGTAAACTTCTAATATCCTCTTTTCTTTTTTCTGTTTATTTTTCTTTGTCTTTGATTGGCCCTTTTCTTATATTGATTTTTGATGTGTTTGATCAGGGACATCACCACAACATTCCCAAGCTTTGTATTCCATTCACAAGTAGATGATACTTGA

CDS

ATGTTGCAGTGCTTAGAGGGATTTAAGTCTTTACTCGCTTCTGTATTGCGTTGTTGTGACATTGATATATATAACCAGGCAAGAGGCCTTGAAGATCCTGAACTTCTTGCAAGGGAGACCGTGTTCAGTGTTAGCGAAATAGAAGCACTGTATGAGCTCTTCAAAAAGATCAGCAGTGCTGTAATCGATGATGGGCTGATTAACAAGGAAGAGTTTCAATTGGCATTATTCAAGACGAATAAAAAAGAAAGTTTGTTCGCTGATCGGGTATTTGATTTATTCGACACAAAGCACAATGGGATCCTCGGTTTTGAAGAGTTTGCTCGTGCCCTATCCGTTTTCCATCCAAATGCCCCTATTGATGATAAGATTGAATTTTCCTTTCAACTATATGATCTCAAGCAGCAGGGTTTTATCGAACGACAAGAGGTGAAGCAAATGGTGGTTGCTACCCTTGCAGAATCGGGTATGAATCTATCGGATGATGTTATAGAAAGTATAATCGACAAGACTTTTGAGGAAGCAGATACAAAGCATGATGGCAAGATTGACAAGGAGGAGTGGAGAAACCTTGTTTTGCGCCATCCGTCACTTTTGAAGAACATGACCCTTCAATATCTCAAGGACATCACCACAACATTCCCAAGCTTTGTATTCCATTCACAAGTAGATGATACTTGA

Protein

MLQCLEGFKSLLASVLRCCDIDIYNQARGLEDPELLARETVFSVSEIEALYELFKKISSAVIDDGLINKEEFQLALFKTNKKESLFADRVFDLFDTKHNGILGFEEFARALSVFHPNAPIDDKIEFSFQLYDLKQQGFIERQEVKQMVVATLAESGMNLSDDVIESIIDKTFEEADTKHDGKIDKEEWRNLVLRHPSLLKNMTLQYLKDITTTFPSFVFHSQVDDT

**CaCBL4**

Gene sequence

ATGGGTTGCTATTTTTCAACTTCAAAGAAATCCAAAATTCCAGGTTATGAGGATCCCACTGTTCTTGCTTCTGAGACACCTTGTGAGTATCCATCATCATTAAGTGGTAAATTCTTGTGATTCTGTTCCACCTGATCACTGGATTTATTAATTAAATGAATTACTAAAGCTTCAAATCTATGTAATACAAGTTTTTTTCTTTCTTTGTTTATAGTTACTGTGAGTGAAGTAGAGGCGTTATATGAACTCTACATGAAGTTAAGCAATTCAATTATTGAAGATGGTCTTATTCATAAGGTGAGTTCAAGTTTGTTTAAAATGTTTTTCAGTTTAGATAAAATGTCCTCATTTTAGGTGCCTAATTTCTTTTGTAAAATTTACTTTTGATGCATTTTTTAATCCAGGAAGAATTTCAGCTAGCACTCTTCAGGAATAGAAACAAGAGGAATCTGTTTGCAGACAGGGTTTGTATAAACTGATCCATGATCTAATGTTTAAACTTGTTCTATATTCTTCAGCCTTCGTACTTTACATAATTATAATCTATGCAACACTGACACTTCTATTTAAAATTTTGTTTTGTATTCGAGTGTGTCAACATTTGATACTTACACGATTCAGTACATATAGTTGTATTAAATCACTTTTATTTTTTATTGGGATCTATGTGTCAATGTCAATGCCGCATGTTTATGTTAGTGTTTCTTAGCGTATAATTTGTGATACCAAATTTTTTGTTGATGTTTAATTTCAGTGAAATCTTCATAGATGTATCATTCTGTTACACGTTGGCACATAATTTCTTTAGACATATCTAGCGCCTTATTATCTCAGTCTAATTGTTTCTCCTTTTATAGATTTTTGACTTATTTGATGTCAAGCGCAACGGGGTTATTGAGTTTGGTGAGTTTGTTCGATCGCTTGGTGTTTTTCATCCAAATGCACCTTTAGAAGACAAAATTGCATGTAAGATTCATTCTCAATTTAACACATAGATTTCATGTTAATGTTTTTTTTTCCTTTCCCTGAGTTTTGGAATGTCCTATAGTAAGCAAGAATTGTATGCTCTACTTATTATTTATTGAATTGGGAGTAATGATTTTTGCAGTTGCTTTTAGGTTGTATGATCTGAGACAAACAGGGTACATTGAAAGAGAAGAGGTAACTTTTTTTTTATTTCTCATTGTCACAAAATTTTATTAGCTTATCCATAAGTCATAGATACATATATATGTGTGTGTGTGTCTTATTATACCCTTTAATTAGTAATCAGTAATTCCACTGCTTATAATCATTGGTTTATCTGCAGTTAAAGGAAATGGTATTGGCACTTCTAAATGAATCAGATCTTGTGCTTTCAGATGACATGATAGAATCAATTGTGGATAAGGTTCATAATATTTTACCTAGTTATTTACGATTATTACATTTTAAAGCTTATTGAAAAATTAGTGTTTCTTAATATAAGTTGTATTGTATCTTCTTCCTCAAGACTTTTAAAGATGCTGATACAAAAGATGATGGAAAGATCGATCAAGATGAGTGGAAAGCATTTGTCACTCAGCATCCATCTTTGATAAAGAACATGACTCTTCCATATCTAAAGTAAGTGCAACCTCTCCTGATTTTCCCTAATTAATTGGAATTTCCAAAAAACATAAGTGGATCATTTCGTAGGTTTAGATTTGGATTTGGTGGAGTCGTTATTCTTCATATTCAATATATTATTAACTGTTGGATTGAGATCGGATGACTAAAATTTTAATTTTTAAAAAAATTTTAAAATCTACGTTGAAATTTAAACTGTCCAATTCAATAGTCGTTAACATATTGATTATATGACCGTAGAAAATCTTAATTTGTTAAATTTGAAGACTGAATTAATTTTGAAGTATGGCTCTTCCATGTTTTGTAGGGATATTTCGATGGCATTTCCTAGTTTTATTGCAAGAACAGAAGTTGAAGAGCAAGAATTGTGA

CDS

ATGGGTTGCTATTTTTCAACTTCAAAGAAATCCAAAATTCCAGGTTATGAGGATCCCACTGTTCTTGCTTCTGAGACACCTTGTGAGTATCCATCATCATTAAGTGTTACTGTGAGTGAAGTAGAGGCGTTATATGAACTCTACATGAAGTTAAGCAATTCAATTATTGAAGATGGTCTTATTCATAAGGAAGAATTTCAGCTAGCACTCTTCAGGAATAGAAACAAGAGGAATCTGTTTGCAGACAGGATTTTTGACTTATTTGATGTCAAGCGCAACGGGGTTATTGAGTTTGGTGAGTTTGTTCGATCGCTTGGTGTTTTTCATCCAAATGCACCTTTAGAAGACAAAATTGCATTTGCTTTTAGGTTGTATGATCTGAGACAAACAGGGTACATTGAAAGAGAAGAGTTAAAGGAAATGGTATTGGCACTTCTAAATGAATCAGATCTTGTGCTTTCAGATGACATGATAGAATCAATTGTGGATAAGACTTTTAAAGATGCTGATACAAAAGATGATGGAAAGATCGATCAAGATGAGTGGAAAGCATTTGTCACTCAGCATCCATCTTTGATAAAGAACATGACTCTTCCATATCTAAAGGATATTTCGATGGCATTTCCTAGTTTTATTGCAAGAACAGAAGTTGAAGAGCAAGAATTGTGA

Protein

MGCYFSTSKKSKIPGYEDPTVLASETPCEYPSSLSVTVSEVEALYELYMKLSNSIIEDGLIHKEEFQLALFRNRNKRNLFADRIFDLFDVKRNGVIEFGEFVRSLGVFHPNAPLEDKIAFAFRLYDLRQTGYIEREELKEMVLALLNESDLVLSDDMIESIVDKTFKDADTKDDGKIDQDEWKAFVTQHPSLIKNMTLPYLKDISMAFPSFIARTEVEEQEL

**CaCBL5**

Gene Sequence

ATGGGGTGTTCTTGTACCAAACCGCGATTTCGGCATGAAGATCCGCCGGCAATTCTTGCTGCACAAACTTACTGTAAGTTTTCTTTTCTTCTTTCTAATCACATCCTGGAGTTTGTATTATATGATTTCATTGATGGAACAGAACAATATTGTAGCTTTGAAGTTTTTAATTAATCAACTTTCTTTTTTCCTGTGTCTATGTATATTTTTTCAGTTAGTATTTCTGAAATTGAAGCATTACATGATCTGTTCAAGAAATTAAGCAGTTCTATAGTCAATGATGGGCTAATTAGCAAAGTAAGGTTTAAGAAATTAATAGATTTAAGGTTGATACCATAATTAAAATATGGTAGGACTTATTAGGAGAGGGTAATTATGTAATATTGAAAATGTAGGAAGAATTTCAGCTTGGCTTATTTGGAAGCAGCAAGAAACGAAACCTCTTTGGTGACAGGGTCAGTGTATTTAACCTTCTTAAGAATTTGTTTTCAACTTATTTTAATAACCTCTTCGAAAAAGTTTATATAAAAATAGTTTGATTTTATTCTAAATAGTTTCCGCATAAGTAGTTATATGATTAACTTTTATGCTATAAACACTTATTATTATAACATTATAACAGCCTAAATTTGTGTGCAGGTTTTCCATTTATTTGACTCAAAAAATGATGGTGTGATAGATTTTGGAGAGTTTGTTGAAGCTTTAAGCGTCTTCCATCCAGCAGCACCCCAAGGACAAAAGGCAGCTTGTAAGAGTTCCTCTCTGAAGAGAATGTGCCTTAGGAATTTTGTATAAAATAAAACCTTCATAATCTCTCTTATATATGTGGCAGTTGCATTTCGACTCTATGATATATGGCAAAGAGGCTTTATTGAACCAGATGAGGTATACAATTGGTTTTTGGCTTTTTTTTTTCTTTCTTTCTTTTTATTAAATAAATTTTAGTATTAATCTATCCAAAACTAAGATAATGAAGAATTAAATAACTTAATACAATCAGTATTACATTAGTCTAAGTGAAACTAAACTCCAATTATTTAGGTAAGATTTAGAGTTTGAGTTTTGTAAATGAAAAAATATAGTTGAGAGAAGAGACCTCACTAAAATACATGTTTTATATTATTATTATTATTTAATTAATGTGTATGTATGCATCAAGACTTGACTTTATGTAGTGTCTAATTGCAGAGGCCCTTAAGAAATAGTACTAGACAATTTTTTTAAAAGACAAAAATTAGTAAATTGTTGGATTCTAACCTTAATAAGTATATTTACAACCTTGTGGAATAATAATGCTTTTCAAATTGTGTGATGGAAGCTAAGAGAGATGATAGAGGCACTCCTAAGAGACTCTGATTTGGTTCTTTCTCATGATATAATTGAGGTCATAATTGATAAGGTATTATTTTTTTAATGTTTTTCTTCCCCTCTCTAAGGATTAATTTTATATTTTCATTATGAAAATAACAGAATCTAGAGATTAGGAATTAGTGGTTTAACATATTATCCTCTCTATGATATTGCACAGGCCTTTAAAGAAGCTGATTTAAAAGGAGATGGAAAGCTTGATCCAGAGGAGTGGGAAAAATTTGTAGCTCGGAATCCATCCTTATTGAAGAATATGACAATTCCATATTTGAAGTAATAACCACTTTTCCTTTCATCCCTTTCTTTTCTGTTTTGAATCATATATATACAATAGTTGCATGACACAGCAATGATAGAAACTTCCTACCCATTACGAGGTCTAAAACAAAATTATCAGTCGGGGTCTTTTCAAAATTTAATAACTATAGAAATTTTCTTTAGTAAAATTAATGACGTTTTCAAATATATTTTCTCTATATTTTTCAAAAAATGAAATAAGACATTTCAATGGTTCTACGACAACATAAAAAACTCGTGTAAAATGTTTCATATTTTAATTGAAGAAGAAAATAGTGTGTATACATTGTATAACTCCAATAACTGATGTTAACAATATGTTATTATTTTTTCAATTTATAACTAATATTTCTCTAATTCTTATTAATTTCATTAACCAATTTAGTTGTATTAAAAAGAGTATTAAATAATTTACAATAAAAATAAAATATTTTATTTAATTTATATAAAATTTTATCAGTTTTAGTTTTAAAAATATTAAAATGTGAGGTCTTTTTTTTGCGTTAAAAAGATTCGAAGTCTTTCAATAAATTTTATTTTATTTTTTAAAGAGTGTTTTGGTCTTTTCTAAACTACTAGTTTAGATAACACAAAAATTCTCTTAACAATTTTCATAGTCATAGTGTTTTTTCGATTGCTATATATTCATTATGTGGAATAAAAATATCATCCAGTTTAAATATCAAAATTGCATATAATTCGGTTCAATTTGAATTGAATTAGAACAAATGATTAAAATAAATAATATATATATATATGGAATTTTGGCATATTGTTTTCTAAAGCAATACATTAACCATTGTTTTTATCGAAAAAATATTTGACATTCACTATTATGAATAACAAAAATTCAAAAAAAACCAAATTTTATTTTGCTAACACTTTTGATAAATAGTAATTAGTTATTATAATTTTTTAAATGATAAGATAATATTTACTAATTCACTAAATTGGTCTCCCTATTTTAAAAGTCAACAGTTTTGGTCCATAATTTTTTAATTAAAAAATGATGACGTGAAATATTTTAAATAACTTGACATTTGATATTATGATTAACACCTATAAAATTGAAATAAAATATGTAAAAATTTAGCTAATAGAACCGATTGCATGAATTGATAATAATAGATGGAATAAAATTCCAATGAAACCTATTATTCAAAAACTAAATGCCCTATATTTTGAATCGCGGAAGATTCCATAGAAAATTTCATAATATATTAAGTGCATACAATGAAATAAAATATTTTTAACTTTCTATCATCTAAGTTTTTTTTTTTCTTATTTGTGGTGGCTTTTCAAATTATAAGCAATAAACATTTCACTTTGAAATTTGCAGGGATCTAAATATGCAGTTTCGTGGTTTTGAATTGATATCAGACATTGAAGATGACACAATCAGTACTCCATGA

CDS

ATGGGGTGTTCTTGTACCAAACCGCGATTTCGGCATGAAGATCCGCCGGCAATTCTTGCTGCACAAACTTACTTTAGTATTTCTGAAATTGAAGCATTACATGATCTGTTCAAGAAATTAAGCAGTTCTATAGTCAATGATGGGCTAATTAGCAAAGAAGAATTTCAGCTTGGCTTATTTGGAAGCAGCAAGAAACGAAACCTCTTTGGTGACAGGGTTTTCCATTTATTTGACTCAAAAAATGATGGTGTGATAGATTTTGGAGAGTTTGTTGAAGCTTTAAGCGTCTTCCATCCAGCAGCACCCCAAGGACAAAAGGCAGCTTTTGCATTTCGACTCTATGATATATGGCAAAGAGGCTTTATTGAACCAGATGAGCTAAGAGAGATGATAGAGGCACTCCTAAGAGACTCTGATTTGGTTCTTTCTCATGATATAATTGAGGTCATAATTGATAAGGCCTTTAAAGAAGCTGATTTAAAAGGAGATGGAAAGCTTGATCCAGAGGAGTGGGAAAAATTTGTAGCTCGGAATCCATCCTTATTGAAGAATATGACAATTCCATATTTGAAGGATCTAAATATGCAGTTTCGTGGTTTTGAATTGATATCAGACATTGAAGATGACACAATCAGTACTCCATGA

Protein

MGCSCTKPRFRHEDPPAILAAQTYFSISEIEALHDLFKKLSSSIVNDGLISKEEFQLGLFGSSKKRNLFGDRVFHLFDSKNDGVIDFGEFVEALSVFHPAAPQGQKAAFAFRLYDIWQRGFIEPDELREMIEALLRDSDLVLSHDIIEVIIDKAFKEADLKGDGKLDPEEWEKFVARNPSLLKNMTIPYLKDLNMQFRGFELISDIEDDTISTP

**CaCBL6**

Gene sequence

ATGGTGCAGTTCTTAGACGTATTGAAGCAACTTTGTGCTGCTGTGGCGAGTTGGTGTGGCGCCGAATTGTCGAGCTCGAAGCAGCCCGGTGGTTTACAAAATCCAGAACTATATTCTAAAGAAACAGTTTGTATGTTATCATCATTGCCATATAACTTGTTTTAGTATCAATACAGTTAGTGTATAACTTGAATATATTTACTAAGCTTCTTGTTAAGGCTTGAGTATAACTTGTTTTAGTATCAATGCAGTTAGTGTAAGTGAGATTGAAGCATTGTATGAACTATTCAAGAAGATCAGCAGTGCAGTTGATGATGATGGACTGATCACTAAGGTTAGTCATCCCATCTCATGCTCAGTGGTTCTACAAGTGTTTTATTAGGCTCATATCTTTTTTGTCCTGCAAATTTACCATTTTTATTGTGGTAAAATGCAGACAAATGTGGTCGATATGGCCACAATTGCGGATGTAAATCGCAATTAACAATCATGGTCCTTACAACAAAAATGATTTTTATAATCCTCATAAATATACTATTTTTCACGTTTGGTCTTTATTATTTAGTTTTAGTCTTTGTAATATTTGTTTATTTCGAAAATTAGTTTTTATAATTTGTGAACCACTTTGATTTTAGTCGTATTTTAGGGACTAAAATCAATATGATGTTATATATTACATAGAAGAAAAAAGATATTTAAACCTTTGGTACGATATTTGTCTGTTAAACATTTTAGAGTGATGGTATTCTTTTCTGCCCCATTGGTATAATCATAAGTGTCTTATTATATGACAGGAAGAATTTCAATTGGCCTTATTCAAGACCAGCAACAAACAAAGCTTGTTTGCAGAGAGGGTATGTAAAAGCTTTCCCTTGTGGTTTTATGTAAAGTGTAAACAAAATATTCTCATGCATTTAGGAAAACTGTTATAATATATCTGGTTAAAATTTACAATGGCAAAAGCAAAGTTGTTTTTTACAACATCTTTTTAGACCTATTTGGCCTATATCACCAAGTTACTCTATAAAGCAATCTATTTTTCTGGTTTATTATCTCTTGATAGCCAATGTTGTAAAATAACGGCTATAGCGGGGCTATCGCGTTATAGCAGAGCGGGGCTATCACGTTATAGCAGAGCGGGGCTATCGCATTATAGTAGAGTGGAATTTGAACAAAATCGCTATTGTTCTGTGATACAATATTTAGTACAAAATATTATCAAATAGCGGCTATAGCGGCCACTTTTGCATAGAGGAATTGGAACAAACTAGTATTTTTTTCGACTCACAACAACATTGTTGATTAGCATTTAGTTATATTAGCGATCCAATGTGTTTGCCTTGTTCATAATTTTACTCTTTTCAAATTATGTAGGTGTTTGACTTGTTTGACACAATGCACCATGGAGTACTTGATTTCAAAGAGTTTGCACTTGCTCTCTCTGTCTTCCATCCTATTGCATCAATGGATGACAAGATTGAATGTAAGATTGACAAATATTTATTAACACTTTAGACATCTTGTAGCCATTAATTTTTATTGTTGATAAGAAAGGAAAGGTTTATTATTGGAGAGGGTATGATCTATTGCTTCTAAAGCTGTTTATTTTTGCTTTCTTATTGCATTTGTTATTGGAAGTAGTTCATAGGCTTTTTGAGGATTCCTAACTTTTACTTCTTGCAGTTTTATTCAGATTGTATGATCTCAAACAGCAAGGATTTATTGAGAGACAAGAGGTAACTTAATTTCTTTTTATATGAAGTTAAAATCAAGTTATAGAATGATGGGGTATTTATCTGATATGCATTTATGATTTCTTTTTGTGTATAGTTGAAACAAATGGTGGTAGCTACTCTATCTGAATCTGGCATGAATCTTTCAGATGAAATGGTTAATACCATCATTGACAAGGTGATTCATTTATTTATCTGATAACTATATCTATGTCAAATCTTATGTTCAGAAGTCTAGGTATATTGAGACATTAATAAGTTCTGTTTCTTTCTTTTTATGTTAAGCTAATCTACAAAATTCATTTTCCTTTTCACTCTTCTTGATAATCTATACAAGTAAAAAAACTTAATTAACAAGCATGCAAGTTATATTCACTGCATTCATACCTTGGAAATGAGAAGTTGGCTTGATGATTATGTAAACTATGCAATGTCGTCAATTGTAGAACGCGGAAAATAGCGATTTGTTCAAATTCCGCTATGCTACTGTGCTATAACTCTGCTTTAGCCTCTATTTGACAATATCTTGTACTAATTAGTGTATTATGGAACAAAAGCAGTTTGTTCAAATTTTGCTACGCTATAGAGTCGTTATAGCCGCTATTCGAGAACACCGAAATTATGCATATGTTGACTTAGCAAAATTATTCTTGTACAATCATAATTTATTTTATAGAGCTATTGATAACAGTTTATAATATATTCAGACATTTGATGAAGTGGACACAAATCATGATGGGAAGATTGACAAAGAAGAATGGCAAAGTCTTGTGATGCAGCATCCCTCTCTTCTGAAAAATATGACTCTTCACTATCTTACGTGAGTTTTCTTCTTGTTGCTATTTTCATCATTTCAGTTTTGCAACTCTGTCAACCAGGGATGAAAGTCTTGCTAGTTTGTTAAATTAA

CDS

ATGGTGCAGTTCTTAGACGTATTGAAGCAACTTTGTGCTGCTGTGGCGAGTTGGTGTGGCGCCGAATTGTCGAGCTCGAAGCAGCCCGGTGGTTTACAAAATCCAGAACTATATTCTAAAGAAACAGTTTTTAGTGTAAGTGAGATTGAAGCATTGTATGAACTATTCAAGAAGATCAGCAGTGCAGTTGATGATGATGGACTGATCACTAAGGAAGAATTTCAATTGGCCTTATTCAAGACCAGCAACAAACAAAGCTTGTTTGCAGAGAGGGTGTTTGACTTGTTTGACACAATGCACCATGGAGTACTTGATTTCAAAGAGTTTGCACTTGCTCTCTCTGTCTTCCATCCTATTGCATCAATGGATGACAAGATTGAATTTTTATTCAGATTGTATGATCTCAAACAGCAAGGATTTATTGAGAGACAAGAGTTGAAACAAATGGTGGTAGCTACTCTATCTGAATCTGGCATGAATCTTTCAGATGAAATGGTTAATACCATCATTGACAAGACATTTGATGAAGTGGACACAAATCATGATGGGAAGATTGACAAAGAAGAATGGCAAAGTCTTGTGATGCAGCATCCCTCTCTTCTGAAAAATATGACTCTTCACTATCTTACTTTTGCAACTCTGTCAACCAGGGATGAAAGTCTTGCTAGTTTGTTAAATTAA

Protein

MVQFLDVLKQLCAAVASWCGAELSSSKQPGGLQNPELYSKETVFSVSEIEALYELFKKISSAVDDDGLITKEEFQLALFKTSNKQSLFAERVFDLFDTMHHGVLDFKEFALALSVFHPIASMDDKIEFLFRLYDLKQQGFIERQELKQMVVATLSESGMNLSDEMVNTIIDKTFDEVDTNHDGKIDKEEWQSLVMQHPSLLKNMTLHYLTFATLSTRDESLASLLN

**CaCBL8**

Gene sequence

ATGTCCATTTCAATGAGCTGCTTTTGCTTGATGAAAAGCAGCAGACCAAAAATTAAGCGAGAACCATTCTCCTTTCTTGCTTTGGAGACATCTTGTAAGTACAAAAATCAACATCTTCCAAATTTTTTGTTATTTTGAACATATGAAGCACTGGCATGACACCGACACGTAAATGCTAATAATAATTTGAGAAAATACAAGTAATTGAAATTAACTATGCGCGTTGTTGTGTTGTGTCACATACACCTTCAATCTAAAATATCAATGCTACATATTACATATTGAACTTGTAATTACAGCCACTTTTTTCAAGCTCTGTTATTTTGAAGTGAAAACAATAAGACAACTTTGTAATTAAAAGTCAAAACAAAAAATAAAAATAGAAAATGCTTGTTCAGGAAACTAAGCAGTTCTTGTGGTTTTAACTCTGAAATGTCCTTTACCATTTGGCAGTTACTGTTAATGAGGTTGAGGCTTTACTTTACTTGTTTAAGAAACTTAGCAGTTCAATTGTTGATGATGGCTGCATTCACAAGGTATATTTATTTTAATAGTAGTAGTTCAAACTAGTTCTAAAGATTTTAAATCTTTTCCTCTATATGAATTGATATAGCATTTCTTCCACATTAACAAGACCTATTTATTTTATTAAATGTCATCACATTTTAGTAGTATTCTTTTTATAAAACATTAGTTTTATGTGCTAATTTTTTTAACACATTTATCTGCAGGAGGAGTTTCAACTTGCACTTTTTAGAAATAGCAGCAAGCAAAATCTACTTGCAAACAGGGTATATAAATGTGTTATTCAAATGTGAATCGCGGTTTATTTCAAATGAAAATTTATTGTGGATTTTCATTTCCTTATAAGTTTTATGCAAACATCTTTCATTTTACTATACATGATGACGTTGAAACTTGCAACCTAAGAAAGCTGAATGCAGGTATTTGATATGTTTGATATTAAGTGTAACGGAGTAATTGAATTTGGTGAATTCGTTCGGTCGCTAAGCATCTTTCATCCAAAGGCATCTGAAGAGAAGAAAATTGAATGTATGAAAATAATTTTGTATCAGAGAAATCAAACACTCATTCATTTCTATGAGATTTTGATTATGTGTCTGCATTAGCCATTCATTCAATTTTTTGTTCTTGCAGTTGCATTTAAATTGTTTGATCTTGGAAAGAATGGATACATTGAACATTGTGAGGTGATTATCCTCTAAGCTTTCCACTGTCTTTCATTTTCTCTTTAGGATTGCAATAATGCAGCACTGGCAATTGTTAGTTTTTTGTGTGAATGCTCCTTAGCATGTAACTTTAGTGTGTGCTTGGTTCTATGCTGGCGAAAATGGATTTTGATTGAATTGATTATGTAAAATTGAGTTTAGTTCAAAGTAGGTTTAGGACAAACAAAGTTATTTATGTTTGGACGCCTTTACATAAAGTTGAGTTGAACATCATTTTAAATGTAAAAATCACGTTTAAAGTAAAAAGTTACAAATGTTAGCTTCACGTTAAAATTAATTTTGAAAGCAAAATCAATTCTAATTAGTACTCTCAAACATACCAAAATCGATCTTAGGTTTCTAGAATCACTTATGACTCTCCCATCAGTGAGTCCAATATACATTTTTGCATTTTGTTTTATTCTACCTTTTTGAGCATCTTATGAGCCTTTTTGTAAAATAATGGCACATTGAAGGTGAAGGAAATGGTCTTGGCTACTTTGACTGAATCAGAAGTTACAATTCCAGATGATATTGTTGAATCCATTGTAGAAAAGGTTCTTAACAATGCAACCATATACATTTAATTTGCCCTCCTTCAATTTCTACATTTTCCTCAATCTAATTCTCATCTCTTATTTTTCTTGTCTTTTATCTTTTTAAGACAATGAAGGAGGTTGACTCAAAGGGAGATGGGAAGATTGATATGGAAGAGTGGAAAGAGTATGCAGAAAAAAATCCTTCTCTTCTTAAGATCATGACCCTTCCATATCTAAAGTAGGTCTAAATCTAATCAATGAAACTTATTGTCTTTATCTAAATTTGATCTCGTTTATTTTCTTTCGAGTAAATCACCATTTAATCATGACATAGTAACTTGGTTAATTTGAGTATCTGAATTTATCAAAGTCTAAATTGATCAACTTAAAAGTTCTAAAGGGAAAGATATTTTCAATTTCATATTTATTCTATTTATTAAATTTCATCTTTTACAGAATCAAAACTCAAATGTTCTGCATTTCAGGGACATAACTCTAGCATTTCCCAGCTTTGTTTTGCATACTGAGGTGGAAGACTAG

CDS

ATGTCCATTTCAATGAGCTGCTTTTGCTTGATGAAAAGCAGCAGACCAAAAATTAAGCGAGAACCATTCTCCTTTCTTGCTTTGGAGACATCTTTTACTGTTAATGAGGTTGAGGCTTTACTTTACTTGTTTAAGAAACTTAGCAGTTCAATTGTTGATGATGGCTGCATTCACAAGGAGGAGTTTCAACTTGCACTTTTTAGAAATAGCAGCAAGCAAAATCTACTTGCAAACAGGGTATTTGATATGTTTGATATTAAGTGTAACGGAGTAATTGAATTTGGTGAATTCGTTCGGTCGCTAAGCATCTTTCATCCAAAGGCATCTGAAGAGAAGAAAATTGAATTTGCATTTAAATTGTTTGATCTTGGAAAGAATGGATACATTGAACATTGTGAGGTGAAGGAAATGGTCTTGGCTACTTTGACTGAATCAGAAGTTACAATTCCAGATGATATTGTTGAATCCATTGTAGAAAAGACAATGAAGGAGGTTGACTCAAAGGGAGATGGGAAGATTGATATGGAAGAGTGGAAAGAGTATGCAGAAAAAAATCCTTCTCTTCTTAAGATCATGACCCTTCCATATCTAAAGGACATAACTCTAGCATTTCCCAGCTTTGTTTTGCATACTGAGGTGGAAGACTAG

Protein

MSISMSCFCLMKSSRPKIKREPFSFLALETSFTVNEVEALLYLFKKLSSSIVDDGCIHKEEFQLALFRNSSKQNLLANRVFDMFDIKCNGVIEFGEFVRSLSIFHPKASEEKKIEFAFKLFDLGKNGYIEHCEVKEMVLATLTESEVTIPDDIVESIVEKTMKEVDSKGDGKIDMEEWKEYAEKNPSLLKIMTLPYLKDITLAFPSFVLHTEVED

**CaCBL9**

Gene sequence

ATGGACCACACTGTATCTCTGGTAATTCAATTTTTATATAACCTTTACATTCGAATTTCTGTTTGATTATAAATCAAATTTTCAATTTATTGTGTTGTGTTGTTTATCAGAGATCGAGTTTGACTTTTGGAGAAACGCTTTGTGCAGTTTTCATTCCACTGATCGGAATAGCTGAAACCTTGTTTTATAGCTTAGCCGGTTGCTTCGATTTCCGTAGTTCAGAGCAGAAGCAGAAAGAGAAGTTCTCTTCTACATTCGACGATTTTCTTGCTCTCGCCAACGATTCTCCTTGTTCGTTTCTCGATTCCTTCCGTTTTCGCTCTTTTTTTTTGTTCAGGAAAAACCTAATTATTATTATTTATGATTTGTGGCAGTTAGTGTGAATGAAATAGAGGCGTTGTTTGAATTGTACAAGAAGTTGAGTTGTTCAATCATTGACGATGGACTAATCCACAAAGTATAGTAATTAACTAATTAACTAACTGCTTTTTTTAATTTGCCTCATATTCTCTCTTTATTTATGTGTAAACCCTAATTCAGTGTTTCAATGTGTAGTCTCATTAGTTTTCCTTTATGGATTCAAGTTTAGTATATATAACCAGCTTAATTAAACACTTATAATGTAAGCACTTATTGTATAAGTGCATATGCATAGACTATTTCTATAACAAATGATGAATTTAAGTCAATCTGTTTTCGTATAAATTGTTTTCATAACCTATCTTTGAGAGCTTACGGACATGTTATAAGCTATTTGTATAAGCTCGCTCAAACAGTCTTGTAAGTGTTTATGACAATAGACAGTTCAATGTAATATTGTAATAATGCAGTAATGAAATAAGGCATTGCTGCATTCATCAGATATGAGTTATTATTGCATAGAAATATCATGGCTCGTATCATGACACCAGCAAATTGGGTTTAATACACAAATATGGAGTCCGTTGGTTCCGATTTGCAGTGAATGAAACTACTCTGTGGTTTCCAATTATTGAATCCAAGTTAACAATTTTTTTCACCAAATTTGCTCCTATAATTTTTATTTGTTTTTATCAAATGCACCTACTATGTGATTCTCATATAGTGCAGTTATGCAGCTAACATCAATGAAATGATGTGAAAATAACAACTTTGATTGTGATCGTTTTTTTTAATCCTAATTGTGTGCTTTTTTTAAAGACAATAATGTGTGTTTGGTGTTGAGTCTGTTAGGCTTATCATTACCACTTACAAGCATAGTGTTGAATTTGTGGTCAGGTTGCACTTGCACCCTTTAACTCTTTTTTCCTCTCTCTCTATGAAGGAAGAGCTTACATTGGCACTATTGAAGACCACAGCTGGCAAGAATCTTTTCCTTGATAGGGTATAAGCATTTTCACAACTGTTATCAATTCTCTTTGTTTTTTGTTTTCTTGATTTCTTTCATGAATCCTTATTGTATTAAGGAAGTTGATTTTTGGTTTGTAAGGAAAACACCTTTCAAGGAGGACCAAATACATTCACCCCCNNNNNNNNNNNNNNNNNNNNNNNNNNNNNNNNNNNNNNNNNNNNNNNNNNNNNNNNNNNNNNNNNNNNNNNNNNNNNNNNNNNNNNNNNNNNNNNNNNNNNNNNNNNNNNNNNNNNNNNNNNNNNNNNNNNNNNNNNNNNNNNNNNNNNNNNNNNNNNNNNNNNNNNNNNNNNNNNNNNNNNNNNNNNNNNNNNNNNNNNNNNNNNTCATGAATCCTTATTGTATTAAGGAAGTTGATTTTTGGTTTGTAAGGAAAACACCTTTCAAGGAGGACCAAATACATTCACCCCCCTGTATTTGGATAAATTGGGGTGAATCTTTATTATGTGGAGATATTTTACTTTCATTTTGTTAGCACAACTCAATCTTTTTATTTTCCTACAAGTTTTCATCCTTTGGAATCTGTAAACCAAAACCCCAAAGCATATTCAATCTGTCCCTTTAAGATTTTTGATCAAGTTGCACCAATACAATCTAATTTTCTCCAATTTCATCTTGAGTCTGTCAAGTTTCAATATGCTTTCTATCTCAGTCTTAAAACTCTGAAGCTGGATTGGTTTCACCAGTATGATTAATTTATGCATTATGCTTTGTTTCAATGGTCTTCTGTTTCTATGTTGACATAGATATATCTGCTCCCAGATGAAAAATTTCGGCTAATAAATTATATGATGATACTCAGGGTTCCAATTATTCATCTTTCCACTCCACATTTTCCCTCTAAAATTCCTTTGGGACATTACGGACTGTTATAGACCGATTGATGATTTAACCAGTCCAATATGTTATGCAATCATCAAGTTCTTGTGCTTTCCATGTCATTCCTTTTGAAGTTGTATAGAGGGATTGGGGCATTAAGTTTGTGATAATCTATCATTTTAGACATATGTGTATACTTGATTTCCAGAAAGATTTGTTTTTTATGGCTAATTGGCAAATCTTCCTTTATGAGCTGTATTATTAAGCTCATTTGATTATTTGTAAAATTCCTAGGTCTTTGATCTCTTTGATGAAAAAAAGAACGGCGTTATAGAATTTGAGGAGTTTGTGCATGCTCTCAGTGTCTTTCATCCTTATACTTCTTTGGAAAAGAAAATTGATTGTAAGACTCCAGTAAGCATTTTGCAGTAGTATTTTTATATTACTAGCCATTTTAATACCATCATCAGTATTGATAAAAATCAATTATAATTTCTTGGGTTGCTTGCAGTTGCTTTTAGATTGTATGACTTGAGACAGACTGGATATATTGAACGAGAAGAAGTGAGTATCTTGGCATGTTTGTGTTACGAATGTGTTATTGGCTCAAACATATTTCTGTTACTTAGAATAGTTTTGCACTAGCATTTTCTTAGTGCAATGCTGATTTTTAATGAAGAAAAGTGTGGTGGTTTGTCCATTGAATAATGTATAAAAACTAAGTTCAAGTAACCACCATGAAGGCTATGCTTTTTTATATAGCATGTTTCCCCTTATGCTTTTATGTTTACCACTGTTTTGTAACTATTTTGTTATCCCACAACTTTTTCCAGGTTCGACAAATGGTGGTTGCCATTTTGTCAGAATGTGGCATGGATGTGGAAAATGAAATCCTTGAAACCATTATTGATAAGGTAATTGTTTGCTGGAACATTTGTTTCTCTCTTATGTTGCATAAGTTTAATCCTCTAACCTACCTTTTTATAAATAAAAAAATCAGACATTTCAAGATGCTGATGCTGACAAGGATGATAAGATCAGTAAAGAAGAGTGGAAAGAATTTGTCATTAAGAACCCATCACTCTTAAGGCACTTGACTCTTCCTAATCTGAAGTAAGATACTCTGATGCCTTACCTCTTTCTCCCATATCTTATATACTCCCTCCATCTCACAATGAGTGTCATTTAATGTTTTTGCACACAAATTAATAAATGAAATAATTAGAAGAAAGAGATAAGTTGTTTTACCAAATTACCCATCTTAATTGTTGGTGAGTTTTTTACTATTATTAGTGCAAAATATAATAATGTTTTAGAAGTTATGTATATAGTAATCATTGAAGGGTAGTATAGGAAAAAATTAATTAAAGTTGCATTAAAAATTGTAAATAACATTCATTTCGAGACAATTTTTTTTGTGCTAAAGTGATACTTATTGTGAAACGGAGAGAATAACTTGTAAAGTTAGTGTTACTTATAAGAGTTGATCATTATATTTTTTACTTCATGGGATGAGTGAATGAGAATGACTTATTGGAGGGATTAGTATCTAAGGTACTAGCAAGTGCTGTATTGTTCAATTCACGACTTGCATAAATGGTCAAGATAAATAAATATTAACATACATAAACTACAGAGAAAGTATTGAAGTTTGAAAGAACATCGTTGTCAGCTCTGCAAACATGTCAAACTAAAAATTGAAGTTGTGTATAAGTCTGTTGGCAAGACTAATAAATTGTTAAACTAATGATTTAAACAAGTTAACTTTGAGTCTTTGACGCATTGCTAATATAAATTTTATTTATACTCGTTTAATCATCAATCCAATTTAAACTCATTCTAGGCACCATTACCAAACTTAGGACAATATTATAATTCAATAGTTTGGACTTTGGATTGCATTGGTTAGGACTGAGTTCAAGCTTTCATTTTTGTTTGGTCATTGGCCAGGGACATAACCACAGTATTTACCAGTTTCATTTTCAACACCGGAGTTGATGATTCCCACTGGCAAGTCAATGGCTAG

CDS

ATGGACCACACTGTATCTCTGAGATCGAGTTTGACTTTTGGAGAAACGCTTTGTGCAGTTTTCATTCCACTGATCGGAATAGCTGAAACCTTGTTTTATAGCTTAGCCGGTTGCTTCGATTTCCGTAGTTCAGAGCAGAAGCAGAAAGAGAAGTTCTCTTCTACATTCGACGATTTTCTTGCTCTCGCCAACGATTCTCCTTTTAGTGTGAATGAAATAGAGGCGTTGTTTGAATTGTACAAGAAGTTGAGTTGTTCAATCATTGACGATGGACTAATCCACAAAGAAGAGCTTACATTGGCACTATTGAAGACCACAGCTGGCAAGAATCTTTTCCTTGATAGGGTCTTTGATCTCTTTGATGAAAAAAAGAACGGCGTTATAGAATTTGAGGAGTTTGTGCATGCTCTCAGTGTCTTTCATCCTTATACTTCTTTGGAAAAGAAAATTGATTTTGCTTTTAGATTGTATGACTTGAGACAGACTGGATATATTGAACGAGAAGAAGTTCGACAAATGGTGGTTGCCATTTTGTCAGAATGTGGCATGGATGTGGAAAATGAAATCCTTGAAACCATTATTGATAAGACATTTCAAGATGCTGATGCTGACAAGGATGATAAGATCAGTAAAGAAGAGTGGAAAGAATTTGTCATTAAGAACCCATCACTCTTAAGGCACTTGACTCTTCCTAATCTGAAGGACATAACCACAGTATTTACCAGTTTCATTTTCAACACCGGAGTTGATGATTCCCACTGGCAAGTCAATGGCTAG

Protein

MDHTVSLRSSLTFGETLCAVFIPLIGIAETLFYSLAGCFDFRSSEQKQKEKFSSTFDDFLALANDSPFSVNEIEALFELYKKLSCSIIDDGLIHKEELTLALLKTTAGKNLFLDRVFDLFDEKKNGVIEFEEFVHALSVFHPYTSLEKKIDFAFRLYDLRQTGYIEREEVRQMVVAILSECGMDVENEILETIIDKTFQDADADKDDKISKEEWKEFVIKNPSLLRHLTLPNLKDITTVFTSFIFNTGVDDSHWQVNG

**CaCBL10**

Gene sequence

ATGCCCACGGATTCCCCTGTAAGTATTTTTCTTCATTTCATTATGACTTTCTAATTATAATTCATGCCCAATAATTAATTAATTGTATGTTTATTGAACAGGGTGGTTCAAGTACAACAATAGGGGAATGTATTTATGCGGCGTTGATGCCACTCATAGCTGTCGTCGAGGTTCTGGTTTTTGCTGTCGCAGGTTGTTTCAATTCTCATCCTCCACTCTCCATTTTTCGCAATCGCAAATCTGCTTATACATCTAATGACTTCGCGCGCCTTGCTGAAGAAACCAGATGTAATGTCACTCATTCCTTTTACTTTTTATTTCAATTTTGTTGTTCAATTGAATCAAAATAATACAATTTCCTTTTTTATTTTTTCAATTTTCAATTTTCATTCATTAGTTCAATTTCAAATTTTACTACTGTATGTTATTTTTTAATATTACATATTTTGACTGAAGTTGACATTTTAACATTGCACATTTTATGATGGGGGTGGATGAAACTTTAGAATCCAAGCATTGTAAAGGATTTAAATTTGCCTGCACCGACAATATCAAATATTTACTTAAGTGTTTACCAAGTCAAGTTTCTCTTCTTATTTGTTCACCGAGAAAACTGTGGTTTTGAGTTATTTTTTGACTTAGAATTACCTATGTATTTATTTTTCCTGGTTTTCATCTAAATAAGTGGTTTTCCTATTTACTATTTTTTTTTGTTTCGTGATTTTGTTGTCTCAAATTCAATTGTTTTATTTTCCGTTTTGGTTAGTGTTTGTTTGATTTCTCATCTTATTGCGGTATTGTTTGATTTAAATTGATATAAACTTCAATCAATTACTGATTCAACTAATGACTATGTCATCAACGTTGCAGATACGAAATCATGAGTATTCTGATTACTTTGATTTTGTCAAATTATTTATGTTTGTAGATAATTTTTTTTTATTTTTAGCAATGTTGAAAATGAATTTTGTTTTCTTTAAGTGTTCAGCCTAAATTTGTTTTATAAAAAAATGTTACACATTTAGCGTGTTCTGAAAGTCAGAATTGAATGAAGATCTGATAATTCATACTTTAATTTCAATAATTCATATTTAAAATTCGTCGTTATGGTTCTGATCAACTGAGTGGTGTTATGAGTGCGGCAAATTTATTTCCAATAATATGCTATAGGTGTTCTGTTTGTTATTATTTTATAATAATGATGATGATATGCACATGTTAAGATCATAAAATATTACATGATTGTAATAGATTCTCTTTCACGAGAAAAACAGCTATATAATTAAGTAAATAATAATAATACATCTAACAGAGCCGACTTTGTTTACTCTAATGGAGTACTCTTATATATTTGTTATTACTTATTAAAACTTTATGCAAGTGGTGTTTCAAGCCAGCTTTGACGGGTCCTAATATGATCTAGTATTAAATTTGTTGAAGCTGTGGAATTTTAGAGAAAATGAGGAGAGAAAATAATAAGGTTTGAATATTATTGATAATAGTTTTATGTTAGTTTCATTACAAAAGACTTAATACTAATCATAATAATAATGAAAGATATTCTAAGATATGATTATAAATTGATCATAGAAAATAACTCAAGATACTCTAATATAATATAAAAGATATTATAAAATATTTTCTAATATTTTCTGACACTCTCCCTCAAGTTAAAGCTTACAAAAAAATAATATGTTACTTTACAAATAAAAAGGATGGAAAGGCAATTCAGGGATGCAAGTGAAGTAAGAGAGAATTGTTATAAATATAACGTAGCCACATAATTGAAATGATCATCAGCATGAGAGAAATTCATGGCAAGTAATTGAATAAAACAAGGTCTGTTGTTCTAAAAGAAATTGCATTTGAAAGTTTCAAATCAATAATATCGGAAACTCAAAATATTTAAACTAGAATATGCTTCAAGGAGAGAGCGACATGATTCAGGAAAAGAAAGGAAAGACCACAGTACATCTGGGACAAATTGCCAAGATAAAGGAGTCATGAAAATAAAAGACATTGTCAGAATAACCATCAACGGAAGAAGAAATCATCACTAATATGATTCATATGTAGGAAAAGGGACACCACCAGAATGATCGTCGATGAGAATAGATGTCACTGTTATAATCTATTAGTAAGAACTAAATTGCATGACAAACACCAAAGAAGAAGCAACGCAACTCTAATGAAACGAAACCATGATTGATCAACGGAGTGAGAAAATGCATCCAAAACAGGAGAGACAATAGTTGTTTGAACAAGGTCGCATATTTTTCGTTGATCAAAATTGAAAAGTAAGGGCAGATCTGGAAACAAGAAGGAAAACCCAATCTAAGATGACTGAAACATTGTGCCGGAAGGCGGATAGACGCGCCTAAGCTCGAGGCTGTTGGGGGGGCGCGTGGAATGAACACAGAGGATCTTGAAGGCACATGAGCGCACGTGGAGCAACATTTGGCATAAAAAATATGGGTTGTGTAGATTGAGAGATTCTGCACACAATGGAAGTGGTTGTGTCAGAAAACTTTAAGCAGCGATAGCAGATGGAAGGCAGCAGTAGAATCTTGAAGGTAGTAGGTGTAACCGTAGCCCGAAACTAGAATACGAGGGCAGATTCGAAACCACGAGGTTGAGGCGCGATTGACGGACCAAGCAGCAACTGAAAAACGTCAAAATGCGCTGTGGAGACGACGACGAGTGAGATCTATTCGCAAGAGAGATTAGCAGCGCGTGAGAGTGCATGTGATGGCTTTTGGCGACGCGCTTTCGGGCATGACCTGATCTAGAAATACTGGCAGCAGCGGCAATAGCGAACTGAACGGAAAACGAGTGAAACGTGTCGGAATGTTGGAAAAGAGAAACTATGATTATATTCCCTAACTGTTGGGAACTCGGCAGCAGAATAGAGGCGGGATCGTTCAAGGAGGATCGAAATTGGCTCTAATACCATGTTGAAGTTGTGGGAGTTTAGAGAAAATGAGAGAAAATAATGAGGGTTTGAATATTTATTGATAATAGCTTTATGCTAACTTCATTACAAAAGACTCAATACTTATTTATTTATAGATAAACATAGACTCAATCCTAAATCAGGAACAAATCATAATAATAATGAGAGATATTCTAAGATATTTCTATGATTATAAATTGATCATAGGAAATAACTCAAGATACTCTAATATAATATAAAAGATATTATAAAATATTTTCTAATATTCTAACATTAAATTTTTTCTAACTACTCAACTTTCTTGTAAATCTATCTGATACTACCTTTGTCCGTCTTAAATTATTTTCTCTCCCCAAGTTACATCTTACATGGGTAAAGTAATTTTATCAAAAAAAGAAAAACAGATGGGTAAAGTAAACTTCTCAATTTGAAGTAAAAGTTTACCTTTTTAAAGTTTCAATCACTTCTAATTATGCTCTATCTCTTATAAGACACACTTTTAAACTGTATAATTATCCATTTGCTGGTTTGAATTTTGAAGAACAATAATAAGTTCAAATAATTGGCTTTTGTGCTGTTAATAATGCAGTTACTGTTAATGAAGTGGAGGCATTGCATGAGCTGTTTAAAAAGATAAGTAGCTCCGTGATTGATGATGGCTTGATTCACAAGGTTAGTTCAATCTGTCAAGTTGTTTGGGTATCTTACAATATCACTAGTCACGTAGTTTAAGTACCTTGAATTTATCATACAAATATATGAAGAGATAGAGGGAGATTCGAATAATCGAATTCAAGTTGGATAGCTGAAATAGAAGAATATTTTTGGTGCATAACATCAGAAAGATGTTATGGTAGCGAATGTTTGGTAATAAATGAACTACTAGAATATAAGAAAAATGTAATGTAAGAAAAGGGTTATAGAGATGAGAATTGTGTTAGATGAATGGTGATGAATGAAAAATGTAATGCATCAGAGAAAATTGGGGTGGCATCTTTTGAGGGAAAACATGGTAAACCTTAATTGGGAGCTAAAAATTTAGTCATTTTCATACAAAATCAATAGGAAGATAGCAAAGATAATCATTTGGTAAACATTTACATTGAGGATTTCACTTTAGTCAGTGATCATGATGCTTGTACTTTTATTCTTTTGTAGGTAGAGCTTCAATTAGCACTATTTCAAACCCCAAATGGAGAAAATCTTTTTCTGGATAGAGTAAGCAGTTCCTATATTTTTTTAGCCATGACATGTTTCTTCACGTACTAGATCAGAAATGAAGTTAAGTTATAATGGAATTACTTTATATTATAATGACTTTATGGGGAAAACCAATTTGCTGCATTTTAGCTGTAATCTTTGCATAATAGTCTATGGTTCATTGATTTGAGATGATATGAACTTTGGCTAATTGGTGGAAACAAAATAGATCCTCTGCAGTCGGCTTTATAGTGCAGTCGGCTGTAGAAGATCCAAGTCCCATGATAGCAAACCATAGCTTAAATTGAAATCACATATTCTATCTCCTTTTCTGTTCCATCAATAGAAACAAAACATCGAAGTAAAAAATAACAATTAAATTCGAGAGAATGTTACTTTTAAATAGACAAAGACAATTTTTAAGGTGTTGTTTTTGAAGAGTCCAATAAGACCATCGCTATTCTTCCAATGATGCCATGAATGTACTACTAATGTTGTGTTAAACTCCATGTCTGTTCTTTAGGTTTTTGATATTTTTGATGAGAAGAGAAATGGTGTTATCGAGTTTGACGAATTCGTCCATGCGCTCGGTGTTTTCCATCCTTATGCCCCTATGGATGAAAAAATAGATTGTACGTTCAAATTGAAAAGAATCTTTTAGTTTCATGCAAAGAAACTCAATTTGTGTTCATACCTAATTGTCTTTGTCCCATAATGATAATTTGCAGTTGCATTTAAGCTCTATGACCTGCGACAAACCGGATTTATTGAGCCAGAAGAAGTGAGTTTACTAATTCATTTAAGTCACATCATTAAGTATTATAAGTTTATTTAAGTGTTTGGTTAACTTGGTTTCAGGTCAAGCAAATGGTAATAGCTATTTTGATGGAATCTGAAATGAATCTTTCTGATGATCTTCTTGAAGCTATTGTTGACAAGGTCAGTTTCTAGTGAAACATGAAAATGTATCATCAATGGAAGATATTGATTATAGGACCTTATCTTTGTCTGCAGACAATTGCAGATGCTGATCAGGACAACGACGGAAAAATCAGTAAAGAAGATTGGAAAGCTTTTGTGAATCGAAATCCATCTCTCTTGAAGAACATGACACTTCCTTATTTGAAGTATGCGAAAATTCCTTTATGTGTGACCGCTCGTTCTTAGTTGGATCTAACTCTTTTAAAGTGAAACAACTCATATAACTCGCATAAAGTGTTACGTGAAATATCAATGAATGATATTATATGGACATGCATAATAAATCATGAATAAATTAAAATATCAACTATTGATTTTTTTCTCTTGTCAAATGCTTCATACTTCTATTCTCCTAGAAGTCTTTAAAACTATAACTAATTGTGTCATTTTGTCATTTTGCAGGGATATCACAACTGCGTTTCCTAGTTTTATTTTCAAATCAGAGGCTGAACTTTGA

CDS

ATGCCCACGGATTCCCCTGGTGGTTCAAGTACAACAATAGGGGAATGTATTTATGCGGCGTTGATGCCACTCATAGCTGTCGTCGAGGTTCTGGTTTTTGCTGTCGCAGGTTGTTTCAATTCTCATCCTCCACTCTCCATTTTTCGCAATCGCAAATCTGCTTATACATCTAATGACTTCGCGCGCCTTGCTGAAGAAACCAGATTTACTGTTAATGAAGTGGAGGCATTGCATGAGCTGTTTAAAAAGATAAGTAGCTCCGTGATTGATGATGGCTTGATTCACAAGGTAGAGCTTCAATTAGCACTATTTCAAACCCCAAATGGAGAAAATCTTTTTCTGGATAGAGTTTTTGATATTTTTGATGAGAAGAGAAATGGTGTTATCGAGTTTGACGAATTCGTCCATGCGCTCGGTGTTTTCCATCCTTATGCCCCTATGGATGAAAAAATAGATTTTGCATTTAAGCTCTATGACCTGCGACAAACCGGATTTATTGAGCCAGAAGAAGTCAAGCAAATGGTAATAGCTATTTTGATGGAATCTGAAATGAATCTTTCTGATGATCTTCTTGAAGCTATTGTTGACAAGACAATTGCAGATGCTGATCAGGACAACGACGGAAAAATCAGTAAAGAAGATTGGAAAGCTTTTGTGAATCGAAATCCATCTCTCTTGAAGAACATGACACTTCCTTATTTGAAGGATATCACAACTGCGTTTCCTAGTTTTATTTTCAAATCAGAGGCTGAACTTTGA

Protein

MPTDSPGGSSTTIGECIYAALMPLIAVVEVLVFAVAGCFNSHPPLSIFRNRKSAYTSNDFARLAEETRFTVNEVEALHELFKKISSSVIDDGLIHKVELQLALFQTPNGENLFLDRVFDIFDEKRNGVIEFDEFVHALGVFHPYAPMDEKIDFAFKLYDLRQTGFIEPEEVKQMVIAILMESEMNLSDDLLEAIVDKTIADADQDNDGKISKEDWKAFVNRNPSLLKNMTLPYLKDITTAFPSFIFKSEAEL
