# Supplementary material for: Investigation of Genes Encoding Calcineurin B-Like Protein Family in Legumes and Their Expression Analyses in Chickpea (Cicer arietinum L.)
Source: PLoS One. 2015 Apr 8;10(4):e0123640. doi: 10.1371/journal.pone.0123640 (PMC4390317; doi:10.1371/journal.pone.0123640)

S2 Fig. Multiple sequence alignment of CBL genes from various plant species.

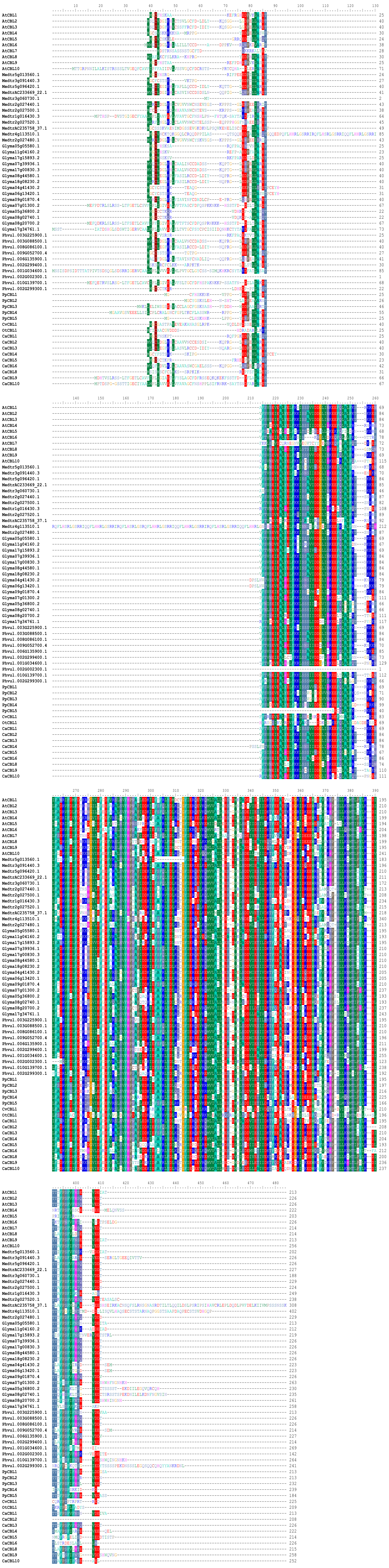

Supplement: S2 Fig — (PDF) [file pone.0123640.s002.pdf]
